# Conditional approval of oncology medicines in Europe (2017–2022): evidence maturity, regulatory outcomes, clinical benefit and downstream access

**DOI:** 10.3389/fphar.2026.1959449

**Published:** 2026-10-13

**Authors:** Mikel Urroz-Elizalde, Paloma Moraga-Alapont, María Jiménez-González, Alberto M. Borobia, Antonio J. Carcas-Sansuán

**Affiliations:** 1 Clinical Pharmacology Department, La Paz University Hospital, IdiPAZ, Madrid, Spain; 2 Department of Pharmacology and Therapeutics, School of Medicine, Universidad Autónoma de Madrid, Madrid, Spain

**Keywords:** antineoplastic agents, clinical benefit, conditional marketing authorisation (CMA), ESMO-MCBS, post-authorisation obligations, reimbursement

## Abstract

**Background:**

Conditional marketing authorisation (CMA) is a European regulatory pathway that enables earlier access to medicines for serious conditions while allowing additional evidence generation after approval. Important questions remain regarding the maturity of supporting evidence, subsequent regulatory outcomes, clinical benefit, and patient access. We characterised the evidentiary basis, regulatory outcomes, clinical benefit, clinical practice guideline incorporation, and downstream access of oncology medicines granted CMA between 2017 and 2022.

**Methods:**

We analysed all antineoplastic medicines granted CMA by the European Commission between 1 January 2017 and 31 December 2022. European Medicines Agency documentation was reviewed to assess regulatory characteristics, post-authorisation obligations, pivotal studies, and outcomes through 31 March 2025. Clinical benefit was evaluated using ESMO–MCBS, while reimbursement was assessed in Spain as an illustrative case of downstream access as of 15 December 2025. Kaplan-Meier analysis estimated time to regulatory resolution and reimbursement.

**Results:**

Twenty-six antineoplastic medicines received CMA, representing 55.3% of all CMA approvals. Most pivotal studies were single-arm (92.9%), open-label, and used surrogate primary endpoints, with a median efficacy sample size of 100.5 participants (IQR 78.5–128.0). Thirty-five specific obligations were imposed, with a median time of 3.95 years (IQR 1.73–5.23) from CMA approval to the latest due date. By 31 March 2025, 53.8% remained under CMA, 38.5% converted to standard marketing authorisation, and 7.7% were withdrawn. Median time to regulatory resolution was 4.46 years. Only 16.7% (3/18) demonstrated substantial clinical benefit according to ESMO–MCBS. Most medicines were subsequently incorporated into ESMO or EHA clinical practice guidelines, although only five (19.2%) received an IA recommendation corresponding to their first CMA-approved indication; two additional medicines received IA recommendations in earlier treatment settings. Public reimbursement was granted for 84.6% (22/26), with a median time of 2.38 years.

**Conclusion:**

Oncology medicines granted CMA were frequently supported by limited and immature evidence, while substantial clinical benefit was uncommon and regulatory resolution often required several years. Future optimisation should combine timely access with timely post-authorisation evidence generation, including comparative clinical and real-world evidence.

## Graphical Abstract

**Figure:**
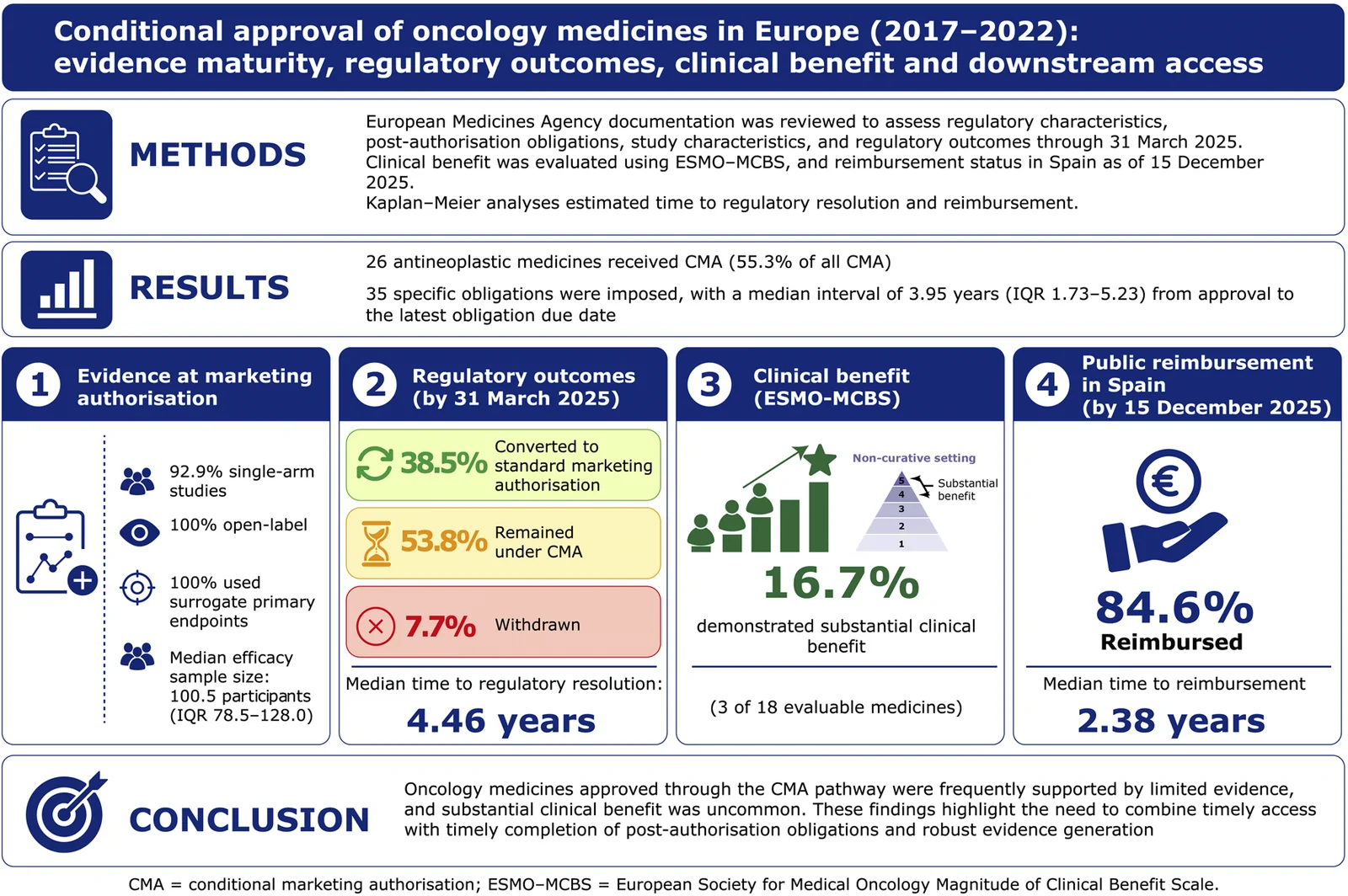

## 1 Introduction

Accelerated regulatory pathways are increasingly used to provide earlier access to medicines for serious or life-threatening conditions with high unmet need, particularly in oncology. In Europe, the conditional marketing authorisation (CMA) pathway, based on assessment by the European Medicines Agency (EMA), allows medicines to be authorised on the basis of less comprehensive clinical data than normally required, provided that the benefit of immediate availability outweighs the risks associated with additional evidence being required after authorisation. CMA is subject to specific post-authorisation obligations and annual reassessment (European Commission, 2004; European Commission, 2006). In the United States, the FDA Accelerated Approval Program similarly facilitates earlier approval of medicines for serious conditions addressing unmet medical needs, typically on the basis of surrogate or intermediate endpoints reasonably likely to predict clinical benefit, with post-approval confirmatory studies required to verify clinical benefit (U.S. Food and Drug Administration, 2026).

Both pathways seek to balance earlier patient access with continued evidence generation after approval. However, reliance on less mature evidence at the time of authorisation may result in uncertainty regarding the magnitude and durability of clinical benefit at the time of approval, creating important challenges for clinicians, patients, regulators, and healthcare systems.

During the first decade of the CMA pathway, approximately half of all conditional approvals were oncology medicines (European Medicines Agency, 2017a). Previous studies have identified important limitations in the evidence supporting conditionally authorised oncology medicines, including frequent reliance on single-arm designs, surrogate endpoints, and relatively small sample sizes. Clinical benefit at the time of approval has also been reported to be uncertain or modest; in one assessment, only 37.5% of conditionally approved oncology medicines were considered to provide high added therapeutic value (Vokinger et al., 2022). Delays in confirmatory studies and prolonged regulatory uncertainty have also been reported. Furthermore, the high costs of many newly authorised cancer medicines create tensions between early regulatory access, subsequent reimbursement decisions, and evidence-based allocation of healthcare resources (Post et al., 2023).

Despite these concerns, the existing literature has predominantly examined the evidentiary characteristics and limitations of oncology medicines at the time of conditional authorisation. Less is known about what happens after authorisation, including how frequently CMA medicines achieve regulatory resolution, the extent to which they demonstrate meaningful clinical benefit, their incorporation into clinical practice guidelines, and whether they ultimately become accessible through public reimbursement. Importantly, these dimensions have rarely been examined together within a contemporary cohort. An updated and integrated assessment may therefore provide a more comprehensive understanding of the relationship between the maturity of evidence at approval, subsequent regulatory outcomes, clinical benefit, and downstream patient access.

We therefore aimed to characterise the evidentiary basis, regulatory outcomes, clinical benefit, clinical practice guideline incorporation, and reimbursement of oncology medicines granted CMA by the European Commission following EMA assessment between 2017 and 2022.

## 2 Materials and methods

### 2.1 Study design

We conducted a systematic analysis of all anticancer medicines granted conditional marketing authorisation (CMA) by the European Commission between 1 January 2017 and 31 December 2022, representing the contemporary CMA era following the first decade of EMA experience (European Medicines Agency, 2017a).

All medicines authorised during the study period were identified from EMA annual reports and regulatory databases. Anticancer medicines were then selected based on the Anatomical Therapeutic Chemical (ATC) classification code L01 (antineoplastic agents) and confirmation of authorisation through the CMA pathway. Detailed data sources are described in the Supplementary Methods.

### 2.2 Data collection

Regulatory and methodological information was extracted from EMA documentation, including the European Public Assessment Report (EPAR) and subsequent regulatory records. Follow-up for regulatory status extended until 31 March 2025.

Extracted variables included regulatory category, orphan designation, scientific advice or protocol assistance, the number and type of specific post-authorisation obligations, characteristics of pivotal studies (study phase, design, blinding, primary endpoints, and efficacy and safety sample sizes), and subsequent regulatory outcomes. The maturity of the supporting evidence was characterised according to study phase, study design, sample size, and primary endpoint. Detailed variable definitions and extraction procedures are provided in the Supplementary Methods.

Clinical benefit at the time of CMA approval was assessed using the European Society for Medical Oncology Magnitude of Clinical Benefit Scale (ESMO–MCBS) (Cherny et al., 2015), based on the pivotal study supporting the first CMA-approved indication for each medicine.

Incorporation into clinical practice guidelines was assessed using the most recent disease-specific European oncology or haematology guidelines available as of 31 May 2026. For each medicine, the first CMA-approved indication was matched with the most recent disease-specific European oncology or haematology guideline available as of 31 May 2026. We recorded the recommendation grade and clinical role and assessed whether the recommendation corresponded to the first CMA-approved indication.

As an illustrative case of downstream access, reimbursement status in Spain for the first CMA-approved indication was obtained from the Spanish Ministry of Health database as of 15 December 2025. Spain was selected as the case country to illustrate the time from European CMA approval to national reimbursement; reimbursement was not intended to represent the situation across European countries.

Data extraction was performed independently by two investigators, with discrepancies resolved by consensus.

### 2.3 Statistical analysis

Characteristics of CMA-approved oncology medicines were summarised using descriptive statistics. Categorical variables are presented as frequencies and percentages, and continuous variables as medians with interquartile ranges (IQRs).

Time from CMA approval to regulatory resolution, defined as conversion to standard marketing authorisation (SMA) or withdrawal, was estimated using the Kaplan-Meier method, with follow-up through 31 March 2025. Time from CMA approval to reimbursement in Spain, considered an illustrative measure of downstream access, was similarly estimated using Kaplan-Meier analysis, with follow-up through 15 December 2025. Medicines withdrawn before reimbursement were censored at the date of withdrawal, whereas medicines not reimbursed by the end of follow-up were censored at the final follow-up date.

The follow-up periods for regulatory resolution and reimbursement were prespecified as 31 March 2025 and 15 December 2025, respectively, independently of the observed time-to-event results. Medicines without an event by the end of follow-up were treated as right-censored. Because more recently approved medicines had a shorter potential follow-up period, they were more likely to be censored. Accordingly, Kaplan–Meier estimates should be interpreted with caution for medicines approved later in the study period because their shorter potential follow-up increases the likelihood of right censoring.

Exploratory multivariable logistic regression was performed to examine methodological characteristics potentially associated with subsequent conversion from CMA to SMA among all oncology medicines approved through the CMA pathway between 2006 and 2022. Prespecified variables included trial phase, study design (single-arm versus multi-arm), and sample size of the primary efficacy population, selected *a priori* as indicators of evidentiary maturity. The period of CMA approval (2006–2016 based on previously published data (European Medicines Agency, 2017b), versus 2017–2022) was included to account for potential temporal changes in regulatory practice. Medicines that remained under CMA or had been withdrawn at the time of analysis were classified as not converted to SMA. Given the limited sample size and number of outcome events, regression analyses were considered exploratory and intended to identify potential associations rather than support causal inference.

All statistical analyses were performed using R version 4.5.1 (RStudio, PBC, Boston, MA, USA).

## 3 Results

### 3.1 Conditional marketing authorisation in oncology

Between 2017 and 2022, the European Commission authorised 530 medicines for human use, of which 47 (8.9%) received conditional marketing authorisation (CMA). Antineoplastic medicines accounted for more than half of all CMA approvals (26, 55.3%).

Compared with 2006–2016, both the number of oncology CMAs and the proportion of newly authorised oncology medicines receiving CMA increased. The annual number of oncology CMAs peaked in 2021, when 10 of 27 newly authorised oncology medicines (37.0%) received conditional approval (Figure 1).

**FIGURE 1:**
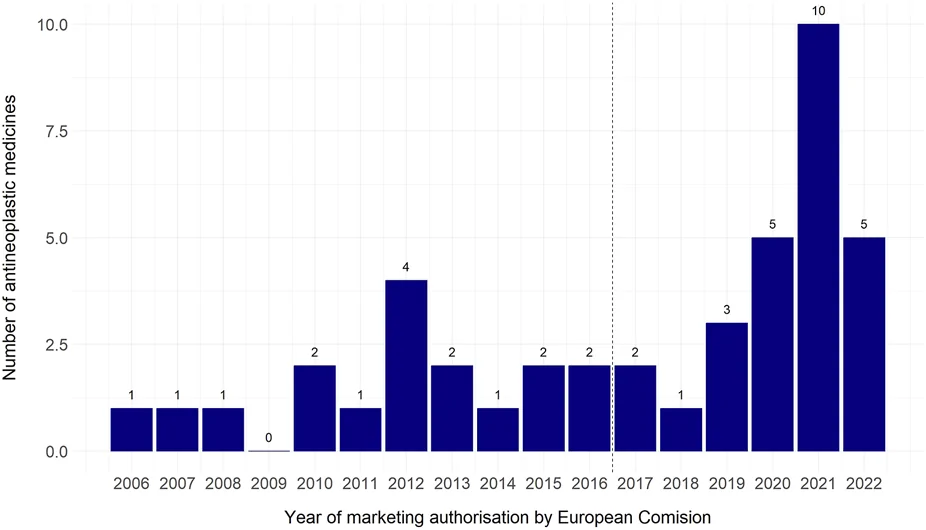
Annual number of antineoplastic medicines granted conditional marketing authorisation by the European Commission (2006–2022). Bars represent the annual number of antineoplastic medicines granted conditional marketing authorisation (CMA) by the European Commission. The dashed vertical line indicates the start of the study period (2017–2022). The number of oncology CMAs increased over time and peaked in 2021 (*n* = 10).

By the end of follow-up (31 March 2025), two of the 26 oncology medicines (7.7%) had been withdrawn from the CMA pathway: Belantamab Mafodotin, following a recommendation not to renew the conditional marketing authorisation, and Pralsetinib, at the request of the marketing authorisation holder.

### 3.2 Regulatory characteristics and post-authorisation obligations

All 26 antineoplastic medicines approved through the CMA pathway during the study period were new active substances. Most were authorised for a single indication (24, 92.3%), whereas Selpercatinib and Entrectinib were each approved for two indications.

Fifteen medicines (57.7%) had orphan medicinal product designation. Most CMA approvals (18, 69.2%) followed an explicit request for conditional authorisation within the initial marketing authorisation application, whereas the CMA pathway was adopted during regulatory assessment for the remaining medicines.

Scientific advice or protocol assistance related to the CMA pathway was obtained for 23 medicines (88.5%). Only three applications proceeded without documented regulatory scientific support.

A total of 35 specific post-authorisation obligations were imposed across the 26 medicines with 1–3 obligations per medicine (median, 2). Most obligations involved interventional clinical trials, predominantly phase III studies (21, 60.0%). The remaining obligations comprised phase II or early-phase studies, extended follow-up of ongoing trials, and non-interventional or registry-based requirements.

Median time from CMA approval to the latest specific obligation due date was 3.95 years (IQR 1.73–5.23), with nine medicines (34.6%) having a final specific obligation due date more than 5 years after approval (Table 1).

**TABLE 1:** Specific obligations, timelines, and regulatory outcomes of antineoplastic medicines granted conditional marketing authorisation between 2017 and 2022.

Active substance	Indication at first approval	Date of 1st authorisation	Specific obligations (*n*)	Latest SO due date	Time to latest SO due date (years)	Regulatory status (31 March 2025)	Date of SMA/withdrawal	Time to outcome or censoring (years)	Final SO type
Alectinib	ALK positive advanced NSCLC	16/02/2017	1	30/04/2018	1.20	SMA	01/12/2017	0.79	Efficacy
Amivantamab	Advanced NSCLC with activating EGFR exon 20 insertion mutations	09/12/2021	1	31/03/2023	1.31	SMA	27/06/2024	2.55	Efficacy
Avapritinib	Unresectable or metastatic GIST harbouring the PDGFRA D842 V mutation	24/09/2020	3	01/12/2027	7.18	CMA	31/03/2025	4.51	Safety
Avelumab	Metastatic merkel cell carcinoma	18/09/2017	1	30/01/2020	2.37	SMA	19/08/2020	2.92	Efficacy
Belantamab mafodotin	Multiple myeloma progression after four prior therapies	25/08/2020	2	01/07/2024	3.85	Withdrawn MA	14/12/2023	3.30	Efficacy
Brexucabtagene autoleucel	Relapsed or refractory MCL after two or more lines of systemic therapy including a BTK inhibitor	14/12/2020	2	30/09/2025	4.79	CMA	31/03/2025	4.29	Safety
Cemiplimab	Metastatic or locally advanced cutaneous SCC who are not candidates for curative surgery or curative radiation	28/06/2019	2	31/03/2023	3.76	SMA	01/07/2022	3.01	Efficacy
Ciltacabtagene autoleucel	Relapsed and refractory multiple myeloma, progression after three prior therapies	25/05/2022	2	01/12/2026	4.52	SMA	19/04/2024	1.90	Safety
Dostarlimab	dMMR/MSI-H recurrent or advanced endometrial cancer	21/04/2021	2	31/12/2022	1.69	SMA	07/12/2023	2.63	Efficacy
Entrectinib	1) Locally advanced, metastatic or where surgical resection is likely to result in severe morbidity solid tumours that have a NTRK gene fusion. 2) ROS1-positive advanced NSCLC not previously treated with ROS1 inhibitors	31/07/2020	2	31/03/2027	6.66	CMA	31/03/2025	4.67	Safety
Idecabtagene vicleucel	Relapsed and refractory multiple myeloma, progression after three prior therapies	18/08/2021	2	01/06/2023	1.79	SMA	19/03/2024	2.58	Safety
Larotrectinib	Locally advanced, metastatic or where surgical resection is likely to result in severe morbidity solid tumours that display a NTRK gene fusion	19/09/2019	3	31/03/2027	7.53	CMA	31/03/2025	5.53	Safety
Loncastuximab tesirine	Relapsed or refractory DLBCL and HGBL after two or more lines of systemic therapy	20/12/2022	2	01/10/2025	2.78	CMA	31/03/2025	2.28	Safety
Lorlatinib	ALK-positive advanced NSCLC whose disease has progressed	06/05/2019	2	30/06/2024	5.15	CMA	31/03/2025	5.90	Efficacy
Mosunetuzumab	Relapsed or refractory follicular lymphoma who have received at least two prior systemic therapies	03/06/2022	1	31/03/2026	3.82	CMA	31/03/2025	2.83	Safety
Pemigatinib	Locally advanced or metastatic cholangiocarcinoma with a FGFR2 fusion or rearrangement that have progressed after at least one prior line of systemic therapy	26/03/2021	2	01/12/2026	5.68	CMA	31/03/2025	4.01	Safety
Polatuzumab vedotin	Relapsed/refractory DLBCL who are not candidates for haematopoietic stem cell transplant	16/01/2020	2	01/10/2021	1.71	SMA	24/05/2022	2.35	Efficacy
Pralsetinib	RET fusion-positive advanced NSCLC not previously treated with a RET inhibitor	18/11/2021	2	31/12/2026	5.12	Withdrawn MA	24/10/2024	2.93	Safety
Rucaparib	Platinum sensitive, relapsed or progressive, BRCA mutated, high-grade epithelial ovarian, fallopian tube, or primary peritoneal cancer, who have been treated with two or more prior lines of platinum-based chemotherapy	24/05/2018	1	01/04/2023	4.85	SMA	09/11/2022	4.46	Efficacy
Selinexor	Progression of multiple myeloma after four prior therapies	26/03/2021	1	01/05/2021	0.10	SMA	18/07/2022	1.31	Efficacy
Selpercatinib	1) Advanced RET fusion positive NSCLC and advanced RET fusion positive thyroid cancer following prior treatment with sorafenib and/or lenvatinib. 2) adults and adolescents ≥12 years with advanced RET mutant MTC following prior treatment with cabozantinib and/or vandetanib	11/02/2021	3	28/02/2025	4.05	CMA	31/03/2025	4.13	Safety
Selumetinib	Symptomatic, inoperable plexiform neurofibromas in paediatric patients with NF1 aged ≥3 years	17/06/2021	3	31/12/2027	6.54	CMA	31/03/2025	3.79	Safety
Sotorasib	Advanced NSCLC with KRAS G12C mutation and who have progressed	06/01/2022	1	31/03/2023	1.23	CMA	31/03/2025	3.23	Efficacy
Tafasitamab	Relapsed or refractory DLBCL who are not eligible for autologous stem cell transplant	26/08/2021	3	01/12/2026	5.26	CMA	31/03/2025	3.59	Safety
Teclistamab	Progression of relapsed and refractory multiple myeloma after three prior therapies	23/08/2022	2	01/12/2028	6.28	CMA	31/03/2025	2.60	Safety
Trastuzumab deruxtecan	Unresectable or metastatic HER2 positive breast cancer who have received two or more prior anti HER2 based regimens	18/01/2021	1	01/03/2022	1.11	CMA	31/03/2025	4.20	Efficacy

Drug-level characteristics of the 26 antineoplastic medicines granted conditional marketing authorisation (CMA) by the European Commission between 1 January 2017 and 31 December 2022. The table summarises the first approved indication, date of initial authorisation, number of specific obligations (SOs) imposed at approval, latest SO due date, time from CMA approval to the latest SO due date, regulatory status as of 31 March 2025, date of conversion to standard marketing authorisation (SMA) or withdrawal when applicable, time to regulatory outcome or censoring, and the type of final outstanding SO (efficacy or safety). Medicines remaining under CMA were censored at 31 March 2025.Abbreviations: ALK, anaplastic lymphoma kinase; BRCA, breast cancer gene; BTK, Bruton’s tyrosine kinase; CMA, conditional marketing authorisation; DLBCL, diffuse large B-cell lymphoma; dMMR, mismatch repair deficient; EGFR, epidermal growth factor receptor; FGFR2, fibroblast growth factor receptor 2; GIST, gastrointestinal stromal tumour; HER2, human epidermal growth factor receptor 2; HGBL, high-grade B-cell lymphoma; KRAS, kirsten rat sarcoma viral oncogene homolog; MA, marketing authorisation; MCL, mantle cell lymphoma; MSI-H, microsatellite instability-high; MTC, medullary thyroid carcinoma; NF1, neurofibromatosis type 1; NSCLC, non-small cell lung cancer; NTRK, neurotrophic tyrosine receptor kinase; PDGFRA, platelet-derived growth factor receptor alpha; RET, rearranged during transfection; ROS1, ROS, proto-oncogene one receptor tyrosine kinase; SCC, squamous cell carcinoma; SMA, standard marketing authorisation; SO, specific obligation.

### 3.3 Evidence supporting conditional approval

The 26 antineoplastic medicines were supported by 28 pivotal studies, as some approvals relied on multiple studies or pooled analyses. The evidentiary basis supporting conditional approval was predominantly non-comparative and based on surrogate efficacy outcomes. Most pivotal studies were single-arm (26, 92.9%), all were open-label, and all used surrogate primary endpoints (Table 2).

**TABLE 2:** Characteristics and primary outcomes of pivotal studies supporting conditional marketing authorisation of antineoplastic medicines (2017–2022).

Active substance	Number of indications at CMA approval	Number of pivotal studies	Phase of pivotal studies	Design of pivotal studies	Primary outcomes	Primary efficacy outcomes (Confidence interval)
Alectinib	1	2	II (both)	Single-arm (both)	ORR (both)	ORR 52.2% (95% CI 39.7–64.6)
						ORR overall RE population 50.8% (95% CI 41.6–59.9) ORR chemotherapy-pretreated 44.8% (95% CI 34.6–55.3)
Amivantamab	1	1	I	Single-arm	ORR	ORR by investigator assessment 36.8% (95% CI 28.0–46.4) ORR by BICR assessment 43.0% (95% CI 33.7–52.6)
Avapritinib	1	1	I	Single-arm	ORR	ORR 94.7% (95% CI 82.3–99.4)
Avelumab	1	1	II	Single-arm	ORR	ORR 33.0% (95% CI 22.3–43.8)
Belantamab mafodotin	1	1	II	Multi-arm randomised (different dose of the CMA product)	ORR	ORR 33.0% in the 2.5 mg/kg dose arm (97.5% CI 22.6–44.7) ORR 32.0% in the 3.4 mg/kg dose arm (97.5% CI 22.1–43.9)
Brexucabtagene autoleucel	1	1	II	Single-arm	ORR	ORR 84.0% (95% CI 73.4–91.3)
Cemiplimab	1	1	II	Single-arm	ORR	ORR 49.2% in group 1 (95% CI 35.9–62.5), 43.6% in group 2 (95% CI 32.4–55.3), and 39.3% in group 3 (95% CI 26.5–53.2)
Ciltacabtagene autoleucel	1	1	II	Single-arm	ORR	ORR 84.1% in the ITT population (95% CI 76.0–90.3)
Dostarlimab	1	1	I	Single-arm	ORR and DOR in responders	ORR was 43.5% (95% CI 34.0–53.4). Median DOR in responders was not evaluable at the data cut-off (1 March 2020) with responses ranging from 2.6 to 28.1 months
Entrectinib	2	*2	Pooled of 3 studies	Single-arm (both)	ORR and DOR (both pivotal studies)	ORR 63.5% (95% CI 51.5–74.4), and median DOR 12.9 months (95% CI 9.3 months to not estimable)
						ORR 73.4% (95% CI 63.3–82.0), and median DOR 16.5 months (95% CI 14.6–28.6)
Idecabtagene vicleucel	1	1	II	Single-arm	ORR	ORR 67.1% (95% CI 59.4–74.9)
Larotrectinib	1	*1	Pooled of 3 studies	Single-arm	ORR	ORR 75.0% (95% CI 64.0–85.0)
Loncastuximab tesirine	1	1	II	Single-arm	ORR	ORR was 48.3% (95% CI 39.9–56.7)
Lorlatinib	1	1	II	Single-arm	ORR	ORR 42.9% in cohort 3 B (95% CI 24.5–62.8), 38.7% in cohorts 4–5 (95% CI 29.6–48.5), and 69.5% in cohorts 2–3 A (95% CI 56.1–80.8)
Mosunetuzumab	1	1	II	Single-arm	CR	CR by IRF 60.0% in cohort B11 FL RP2D (95% CI 49.1–70.2)
Pemigatinib	1	1	II	Single-arm	ORR	ORR 37.0% (95% CI 27.94–46.86)
Polatuzumab vedotin	1	1	II	Multi-arm randomised (standard of care as the control arm)	CR	CR 40.0% in arm C (Pola + BR) (95% CI 24.9–56.7) and 17.5% in arm D (BR) (95% CI 7.3–32.8) with an absolute difference of 22.5 percentage points (95% CI 2.6 to 40.2; P = 0.0261)
Pralsetinib	1	1	II	Single-arm	ORR	ORR 58.8% in group 1 (95% CI 50.1–67.2), 72.0% in group 2 without prior systemic treatment (95% CI 60.4–81.8), and 64.4% overall (95% CI 57.9–70.5)
Rucaparib	1	*1	Pooled of 2 studies	Single-arm	ORR	ORR 54.7% (95% CI 44.8–64.4)
Selinexor	1	1	IIb	Single-arm	ORR	ORR in the BCLPD-refractory population 25.3% (95% CI 16.4–36.0)
Selpercatinib	2	1	II	Single-arm	ORR	In the NSCLC cohort, ORR 63.8% (95% CI 53.9–73.0), in the MTC cohort, ORR 69.1% (95% CI 55.2–80.9), In the TC cohort, ORR 78.9% (95% CI 54.4–93.9)
Selumetinib	1	1	II	Single-arm	ORR	ORR 66.0% (95% CI 51.2–78.8)
Sotorasib	1	1	II	Single-arm	ORR	ORR 37.1% (95% CI 28.6–46.2)
Tafasitamab	1	1	II	Single-arm	ORR	ORR with tafasitamab plus lenalidomide 56.8% (95% CI 45.3–67.8)
Teclistamab	1	1	II	Single-arm	ORR	ORR 63.0% in cohort a (95% CI 55.2–70.4) and 52.5% in cohort C (95% CI 36.1–68.5)
Trastuzumab deruxtecan	1	*1	Pooled of 2 studies	Single-arm	ORR	ORR 58.3% (95% CI 51.7–64.7)

* Pooled analyses.

Characteristics of the 28 pivotal studies supporting conditional marketing authorisation (CMA) of 26 antineoplastic medicines granted by the European Commission between 1 January 2017 and 31 December 2022. The table summarises the number of approved indications at CMA, number of pivotal studies supporting each medicine, study phase, trial design, primary efficacy endpoints, and results of the primary efficacy outcomes with corresponding confidence intervals. Some medicines were supported by pooled analyses of multiple clinical studies or by more than one pivotal study for the same or different indications.

Abbreviations: BICR, blinded independent central review; BR, bendamustine plus rituximab; CI, confidence interval; CMA, conditional marketing authorisation; CR, complete response; DOR, duration of response; FL, follicular lymphoma; IRF, independent review facility; ITT, intention-to-treat; MTC, medullary thyroid carcinoma; NSCLC, non-small cell lung cancer; ORR, overall response rate; OS, overall survival; PFS, progression-free survival; Pola, Polatuzumab Vedotin; RE, response-evaluable; RP2D, recommended phase II, dose; TC, thyroid carcinoma.

Only two pivotal studies used a multi-arm randomised design. Only one of these studies (Polatuzumab Vedotin) included a standard-of-care comparator and demonstrated statistically significant improvements in both progression-free survival (PFS) and overall survival (OS). Overall response rate (ORR) was the most common primary endpoint (23, 82.1%). Three studies used ORR and duration of response as co-primary endpoints, whereas two used complete response as the primary endpoint.

Study populations were generally small, with a median efficacy sample size of 100.5 participants (IQR 78.5–128.0) (Supplementary Table S1). PFS was reported as a secondary endpoint in all pivotal studies, although median PFS was not reached in one study. Overall survival was reported in 25 studies. Median OS had not been reached in nine studies at the time of regulatory assessment, reflecting uncertainty regarding long-term clinical benefit (Supplementary Table S2).

For safety evaluation, the median sample size was 411.5 participants (IQR 229.8–538.5). Safety assessments were generally based on pooled populations from pivotal and supportive studies. Only two medicines (Brexucabtagene Autoleucel and Ciltacabtagene Autoleucel) relied exclusively on pivotal trial safety data (Supplementary Table S3).

### 3.4 Regulatory outcomes

As of 31 March 2025, 14 of 26 antineoplastic medicines (53.8%) remained under conditional marketing authorisation (CMA), 10 (38.5%) had converted to standard marketing authorisation (SMA), and 2 (7.7%) had been withdrawn.

The median time from CMA approval to regulatory resolution, defined as conversion to SMA or withdrawal, was 4.46 years (Figure 2). This exceeded the median time to the latest specific obligation due date (3.95 years), indicating prolonged regulatory follow-up after initial approval.

**FIGURE 2:**
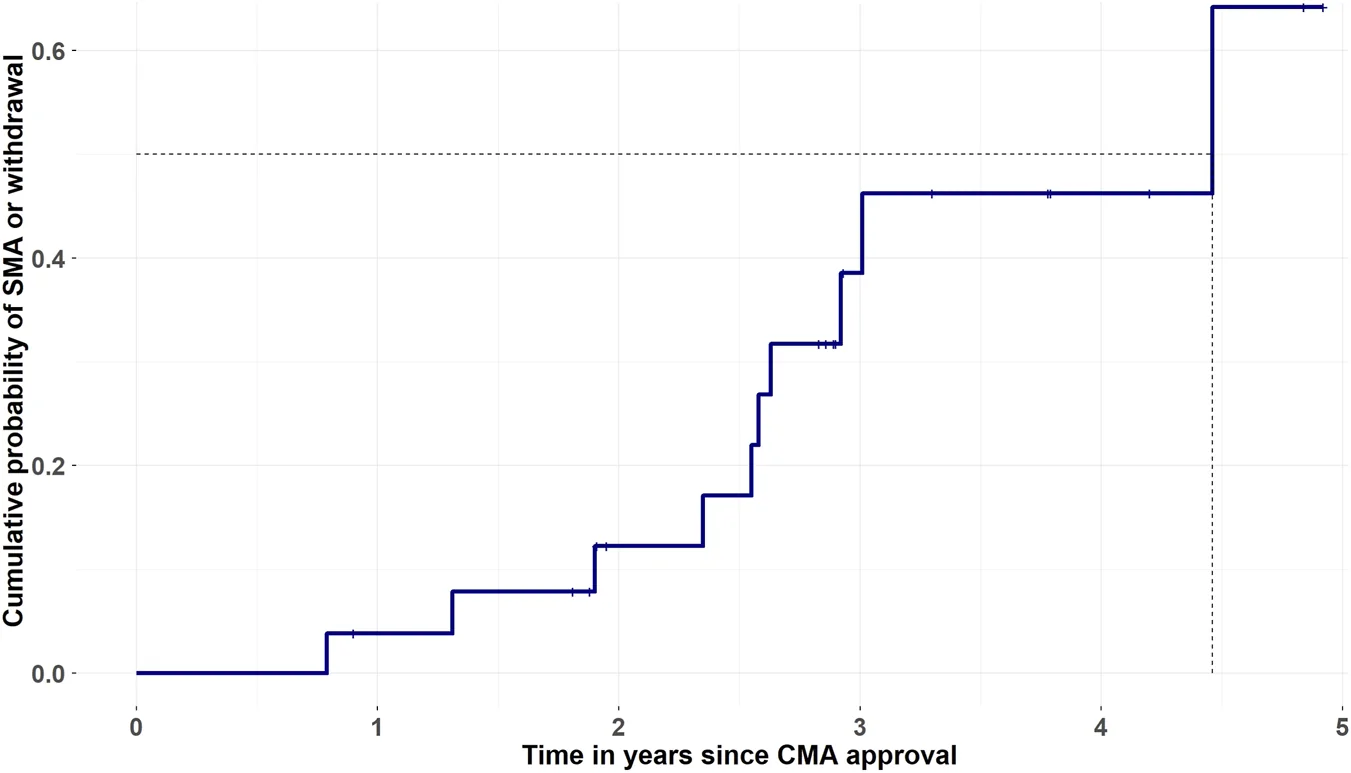
Time from conditional marketing authorisation to regulatory resolution. Kaplan–Meier curve showing time from conditional marketing authorisation (CMA) to regulatory resolution among 26 antineoplastic medicines approved between 2017 and 2022. Regulatory resolution was defined as conversion to standard marketing authorisation (SMA) or withdrawal. Medicines remaining under CMA at 31 March 2025 were censored. Abbreviations: CMA, conditional marketing authorisation; SMA, standard marketing authorisation. The dashed lines indicate the median time to public reimbursement (2.38 years).

### 3.5 Factors associated with conversion to standard marketing authorisation

Among antineoplastic medicines approved through the CMA pathway between 2006 and 2022, descriptive analyses suggested that conversion to standard marketing authorisation (SMA) was more frequent among medicines supported by phase III trials, multi-arm study designs, and larger efficacy populations (Supplementary Table S4). In the 2017–2022 cohort, 10 of 26 medicines (38.5%) converted to SMA during follow-up.

In exploratory multivariable logistic regression analyses, trial phase, study design, sample size, and period of CMA approval were not significantly associated with conversion to SMA (Supplementary Table S5). Confidence intervals were wide, consistent with the limited sample size and number of outcome events. These analyses should therefore be considered hypothesis-generating rather than confirmatory.

### 3.6 Clinical benefit

ESMO–MCBS scores were available for 18 of the 26 antineoplastic medicines. Only three of 18 evaluated medicines (16.7%) demonstrated substantial clinical benefit according to ESMO–MCBS. ESMO–MCBS evaluations were unavailable for eight medicines, including both approved on the basis of randomised multi-arm trials.

### 3.7 Clinical practice guideline incorporation

Most CMA-approved medicines were subsequently incorporated into ESMO or European Haematology Association (EHA) clinical practice guidelines, with recommendation grades ranging from IA to VB (Supplementary Table S6). Five medicines (19.2%)—Alectinib, Ciltacabtagene Autoleucel, Idecabtagene Vicleucel, Selinexor, and Trastuzumab Deruxtecan—received an IA recommendation corresponding to their first CMA-approved indication. Alternative therapies with the same clinical role and recommendation grade were identified for most IA-recommended indications, with selpercatinib being the exception. Lorlatinib and selpercatinib also received IA recommendations for first-line treatment of ALK-positive and RET fusion-positive NSCLC, respectively, representing treatment settings earlier in the treatment pathway than their first CMA-approved indications. This assessment was based on guideline recommendations and did not imply that these alternatives were clinically equivalent or appropriate for every patient. Only one medicine (Mosunetuzumab) was not included in a corresponding ESMO or EHA guideline, while the original CMA indication for rucaparib was not represented in current ESMO recommendations.

### 3.8 Reimbursement in Spain

By 15 December 2025, public reimbursement in Spain had been granted for 22 of 26 antineoplastic medicines (84.6%). Median time from CMA approval to reimbursement was 2.38 years (Figure 3), substantially shorter than the median time to regulatory resolution (4.46 years).

**FIGURE 3:**
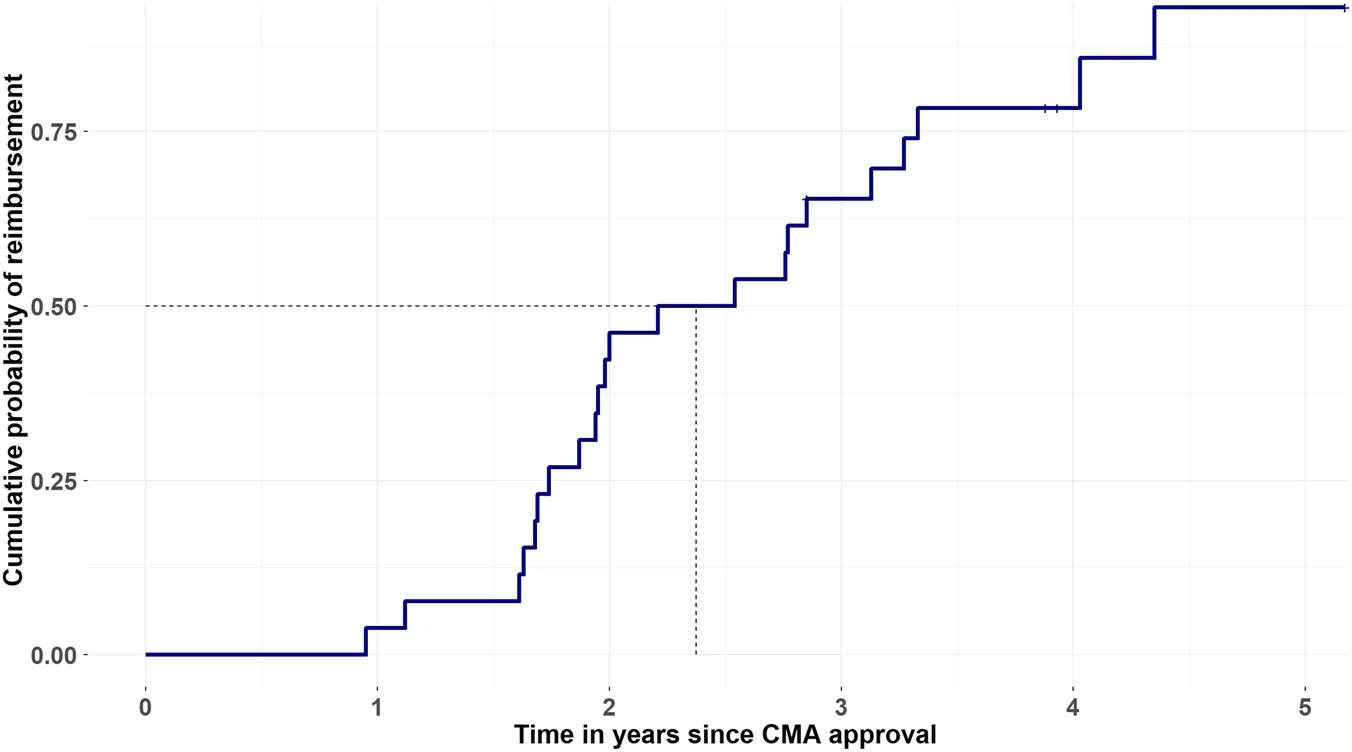
Time from conditional marketing authorisation to public reimbursement in Spain. Kaplan–Meier curve showing time from conditional marketing authorisation (CMA) to public reimbursement in Spain among 26 antineoplastic medicines approved between 2017 and 2022. Medicines not reimbursed by 15 December 2025 were censored at the final follow-up date. Abbreviations: CMA, conditional marketing authorisation.

Reimbursement was observed across medicines with different ESMO–MCBS scores, including all medicines with substantial clinical benefit and most with moderate benefit. Among the four medicines that had not obtained reimbursement by the end of follow-up, two had been withdrawn and two remained authorised but unreimbursed (Supplementary Table S7).

## 4 Discussion

### 4.1 Main findings

This study provides a contemporary evaluation of antineoplastic medicines granted conditional marketing authorisation (CMA) in Europe between 2017 and 2022. More than half of all medicines granted CMA during this period were antineoplastic agents, underscoring the continued importance of expedited regulatory mechanisms in oncology. We found that CMA approvals were predominantly supported by small, open-label, single-arm studies using surrogate endpoints, reflecting limited evidentiary maturity at the time of authorisation. Despite these limitations, most medicines were subsequently reimbursed in Spain, whereas fewer than half had reached regulatory resolution through conversion to standard marketing authorisation (SMA) or withdrawal by March 2025. Overall, these findings illustrate the persistent tension between earlier access to promising therapies and the need for robust evidence to inform subsequent regulatory and healthcare decisions.

### 4.2 Interpretation of the findings

#### 4.2.1 Evidence supporting conditional approval

Compared with the first decade of the CMA pathway (2006–2016), the proportion of oncology medicines receiving CMA remained broadly similar. However, the evidentiary basis for approval appeared less mature in the contemporary cohort, with most approvals relying on non-comparative studies and surrogate outcomes. Recent analyses of oncology and haematology medicines approved through the CMA pathway have similarly reported increasing reliance on conditional approvals and less mature evidence, despite the availability of alternative treatment options in many settings (Lasch et al., 2025). This pattern may reflect broader changes in oncology drug development, including the expansion of precision medicine, biomarker-defined indications, rare cancer subgroups, and cell and gene therapies, for which conventional randomised trials may be difficult to conduct. Comparisons across time periods should nevertheless be interpreted cautiously given changes in regulatory practices and evidentiary standards.

The limited maturity of evidence was particularly evident in study design and endpoint selection. Nearly all pivotal studies were single-arm and all used surrogate primary endpoints. Overall response rate (ORR) was the primary endpoint in most studies, whereas progression-free survival (PFS) and overall survival (OS) were generally secondary endpoints and were frequently immature at the time of approval. Although surrogate endpoints can facilitate earlier access to promising therapies, substantial evidence indicates that they are not consistently correlated with clinically meaningful outcomes such as overall survival (Zia et al., 2005; Haslam et al., 2019). Single-arm studies may also overestimate treatment effects due to selection bias, lack of randomisation, and the absence of direct comparative evidence. These findings are consistent with a recent ESMO Open analysis of anticancer medicines approved in Europe on the basis of single-arm trials, which reported that only a minority demonstrated substantial clinical benefit according to ESMO–MCBS despite regulatory approval, highlighting the limitations of non-comparative evidence for establishing meaningful therapeutic benefit (Mulder et al., 2023).

Real-world evidence (RWE) may complement randomised controlled trials in pre- and post-authorisation settings by providing information on effectiveness and safety in routine practice, particularly when trials are difficult to conduct or uncertainties remain following CMA. Reliable and timely RWE requires pre-established, multicentre data-generation structures and adequate human and material resources. However, limitations including confounding, selection bias, and variable data quality warrant interpretation alongside controlled clinical evidence.

The frequent reliance on non-randomised evidence should, however, be considered in the context of high unmet medical need. Many CMA approvals involved rare cancers, biomarker-defined populations, orphan indications, or paediatric malignancies, in which recruitment for large randomised trials may be impractical or ethically challenging. In these settings, the CMA pathway can expand treatment options and facilitate earlier access to promising therapies for patients with limited alternatives, while allowing additional evidence to be generated after approval. However, specific post-authorisation obligations often extended several years beyond approval, indicating that evidentiary uncertainties accepted at approval may persist for prolonged periods as described previously (Bellino and La Salvia, 2025). Ensuring that these uncertainties are adequately addressed through timely and robust confirmatory evidence generation remains a central challenge of the CMA pathway.

For patients and clinicians, limited evidence at approval translates into residual uncertainty regarding both the magnitude and durability of treatment benefit. Under the CMA pathway, this uncertainty is managed through specific post-authorisation obligations, annual reassessment, and the possibility of conversion to SMA, continuation of CMA status, or withdrawal if the benefit–risk balance becomes unfavourable. Importantly, conversion to SMA indicates that regulators consider the available evidence sufficient to support standard authorisation, but it does not necessarily mean that all clinically meaningful outcomes, including overall survival or quality-of-life benefits, have been conclusively established. Long-term safety and effectiveness may remain under evaluation even after conversion.

Rucaparib illustrates the limitations that may arise when conditional authorisation relies predominantly on surrogate efficacy outcomes. Although it was granted CMA for BRCA-mutated ovarian cancer based primarily on surrogate efficacy outcomes, confirmatory evidence from the ARIEL4 trial failed to demonstrate an overall survival advantage compared with chemotherapy (Oza et al., 2025). This example illustrates how approvals based predominantly on surrogate endpoints and uncontrolled evidence may not reliably predict improvements in long-term clinically meaningful outcomes. Consistent with this concern, validation studies have shown that many surrogate endpoints used in oncology demonstrate only weak or inconsistent correlation with overall survival (Walia et al., 2022). These observations support the use of surrogate endpoints when their ability to predict clinically meaningful outcomes is supported by robust validation evidence (Kemp and Prasad, 2017).

#### 4.2.2 Clinical benefit and guideline incorporation

The limited maturity of evidence at approval was reflected in assessments of clinical benefit. ESMO–MCBS evaluations indicated substantial clinical benefit for only three medicines, while no corresponding ESMO–MCBS evaluation was available for eight medicines supporting the CMA-approved indication. Although ESMO–MCBS is widely used to assess clinical benefit in oncology, its scores should be interpreted in the context of the evidence available at the time of assessment and the limitations of the underlying studies. Our findings are consistent with previous literature. Brinkhuis and colleagues found that many oncology medicines granted CMA had low or non-quantifiable added therapeutic value (Brinkhuis et al., 2024), while Bloem and colleagues reported that clinical benefit often remained limited even after conversion from CMA to SMA (Bloem et al., 2022). Similarly, a recent ESMO Open study evaluating uncertainties in oncology medicines authorised by the EMA found that conditionally authorised medicines had among the highest levels of unresolved regulatory uncertainty, particularly regarding efficacy and safety. Together, these findings suggest that regulatory conversion does not necessarily coincide with complete resolution of clinically relevant evidence gaps.

Most CMA-approved medicines were subsequently incorporated into ESMO or EHA clinical practice guidelines. However, only a minority received the highest recommendation grades, and most medicines with grade IA recommendations had at least one alternative therapy with the same clinical role and recommendation grade. Thus, even when CMA medicines do not represent uniquely transformative treatment options, their incorporation into clinical practice may nevertheless expand therapeutic choice and provide additional treatment options for patients, particularly in settings of substantial unmet need.

#### 4.2.3 Regulatory outcomes

Our findings demonstrate prolonged timelines for post-authorisation obligations and regulatory decision-making. Specific post-authorisation obligations frequently extended several years beyond approval, with a median interval of almost 4 years between CMA approval and the latest due date of imposed obligations. Nine medicines had final obligation due dates more than 5 years after approval. These findings suggest that evidentiary uncertainties accepted at the time of approval may persist for several years.

By the end of follow-up, more than half of medicines remained under CMA, and the median time to regulatory resolution was 4.46 years. Although we explored whether methodological characteristics of pivotal studies were associated with conversion to SMA, no statistically significant associations were identified. These exploratory analyses should be interpreted cautiously given the limited sample size and number of outcome events, and the absence of statistically significant associations should not be interpreted as evidence that these characteristics have no relationship with regulatory outcomes.

#### 4.2.4 Reimbursement and patient access

The relationship between evidentiary limitations and patient access is particularly relevant from a health policy perspective. Using Spain as an illustrative case of downstream access, most CMA-approved medicines received public reimbursement despite generally modest ESMO–MCBS scores and ongoing regulatory uncertainty. The median time to reimbursement was 2.38 years. Similar reimbursement delays have been reported in other European countries (Hammerman et al., 2018; Janzic et al., 2019; Thomson et al., 2022). However, this was substantially shorter than the median time to regulatory resolution (4.46 years), suggesting that reimbursement may occur while confirmatory evidence generation is still ongoing. This pattern suggests that health systems may provide access to medicines before their long-term benefits and risks are fully established.

Reimbursement decisions appeared only partially aligned with standardised assessments of clinical benefit. Factors beyond measured clinical benefit—including disease severity, orphan designation, unmet medical need, cost-effectiveness considerations, budget impact, and societal preferences—are likely to contribute to reimbursement decisions (Nieto-Gómez and Castaño-Amores, 2024). While this may facilitate earlier access to innovative therapies and expand treatment options for patients with substantial unmet need, it also raises important questions regarding opportunity costs and the allocation of limited healthcare resources.

#### 4.2.5 Implications for regulatory policy

Taken together, these findings illustrate the continuing challenge of balancing timely access to innovative cancer therapies with the need for robust evidence regarding their benefits and risks. The CMA pathway is intended to address unmet medical needs while ensuring that residual uncertainties are addressed through post-authorisation evidence generation. However, prolonged timelines for specific obligations, continued reliance on surrogate endpoints, and widespread reimbursement before regulatory resolution raise important questions regarding evidentiary standards and value-based access. Strengthening requirements for robust evidence generation before and after approval, promoting greater use of randomised comparative designs where feasible, and ensuring timely completion of post-authorisation obligations may help maximise the value of expedited regulatory pathways while maintaining public confidence in regulatory decision-making.

### 4.3 Strengths and limitations

A major strength of this study is the comprehensive evaluation of the CMA pathway, integrating evidentiary maturity, regulatory outcomes, clinical benefit, reimbursement, and incorporation into clinical practice guidelines within a contemporary cohort of oncology medicines. Several limitations should nevertheless be acknowledged.

First, the analysis was restricted to publicly available EMA and European Commission regulatory documents and therefore may not fully capture internal regulatory deliberations or ongoing post-authorisation discussions. Second, the relatively small number of oncology medicines approved through the CMA pathway limited the statistical power of exploratory analyses examining factors associated with conversion to SMA. Third, assessment of clinical benefit relied on ESMO–MCBS evaluations available at the time of analysis, and several medicines had not yet been assessed using this framework. Fourth, reimbursement was assessed only in Spain as an illustrative case of downstream access and may not be generalisable to other European healthcare systems, which differ in reimbursement procedures, health technology assessment frameworks, and access policies. Finally, several medicines remained under CMA and continued to generate post-authorisation evidence at the end of follow-up; consequently, some regulatory outcomes may change over time.

### 4.4 Conclusions

In conclusion, oncology medicines approved through the CMA pathway between 2017 and 2022 were generally supported by limited and immature evidence at the time of authorisation, most often derived from single-arm studies using surrogate endpoints. Substantial clinical benefit was uncommon, while regulatory resolution frequently required several years. Future optimisation of the CMA pathway should therefore combine timely access with timely completion of post-authorisation obligations and systematic generation of robust post-authorisation evidence, including comparative clinical and real-world evidence, by marketing authorisation holders.

## Data Availability

The original contributions presented in the study are included in the article/Supplementary Material, further inquiries can be directed to the corresponding author.
