# Supplementary material for: Conditional approval of oncology medicines in Europe (2017–2022): evidence maturity, regulatory outcomes, clinical benefit and downstream access
Source: Front Pharmacol. 2026 Oct 13;17:1959449. doi: 10.3389/fphar.2026.1959449 (PMC13710259; doi:10.3389/fphar.2026.1959449)
Supplement: Supplementary file 1 [file Supplementaryfile1.pdf]

Conditional approval of oncology medicines in Europe (2017–2022): evidence maturity, regulatory outcomes, clinical benefit and downstream access

SUPPLEMENTARY MATERIAL

Supplementary material provides detailed definitions of regulatory and methodological variables, comprehensive efficacy and safety results from pivotal studies supporting conditional marketing authorisation (CMA), and additional analyses exploring factors associated with conversion to standard marketing authorisation.

List of abbreviations

- BICR: blinded independent central review
- BOR: best overall response
- CAT: committee for advanced therapies
- CBR: clinical benefit rate
- CDOR: central nervous system duration of response
- CHMP: committee for medicinal products for human use
- CMA: conditional marketing authorisation
- CORR: central nervous system objective response rate
- CPR: CNS progression rates
- CR: complete response
- DCR: disease control rate
- DOR: duration of response
- EHA: European Haematology Association
- EPAR: European Public Assessment Reports
- ESMO: European Society for Medical Oncology
- IC: intracranial
- ICR: independent central review
- IERC: independent endpoint review committee
- IRC: independent review committee
- ITT: intention to treat
- MAA: marketing authorisation applications
- MAH: marketing authorisation holder
- MKI: multi-kinase inhibitor
- mITT: modified intention to treat

- MTC: medullary thyroid cancer
- NSCLC: non-small cell lung cancer
- ORR: objective response rate
- OS: overall survival
- PFS: progression free survival
- PP: per protocol
- PR: partial response
- QD: once daily
- R/R: relapse/refractory
- RE population: response evaluation population
- RECIST: response evaluation criteria in solid tumours
- REiNS: response evaluation in neurofibromatosis and schwannomatosis
- SMA: standard marketing authorisation
- Scr: stringent complete response
- TC: thyroid cancer
- TTNT: time to next treatment
- TTR: time to tumour response
- VGPR: very good partial response

Abbreviations are reported as used in original pivotal studies and may vary across trials.

Supplementary Methods: data extraction and variable definitions

All medicines authorised by the European Commission between 1 January 2017 and 31 December 2022 were identified using EMA annual reports and regulatory databases:

- EMA annual reports and work programmes: https://www.ema.europa.eu/en/about-us/annual-reports-work-programmes
- EMA medicines database: https://www.ema.europa.eu/en/medicines

For each medicine, information was extracted from:

- Initial Marketing Authorisation Documents (European Public Assessment Reports, EPARs)
- Procedural Steps Taken and Scientific Information After Authorisation
- Changes Since Initial Authorisation of Medicine

1.1. Regulatory variables extracted

- Active substance
- European Commission Decision Date. Date of first authorisation by CMA valid throughout the European Union.
- ATC CODE REVIEWED after last updated on 26 January 2024 (https://atcddd.fhi.no/atc_ddd_index/): 1st to 4th level therapeutic subgroup.
- Application type (Article 8(3) of Directive No 2001/83/EC): New active substance, fixed combination application, generic application, hybrid application, informed consent application, known active substance, similar biological application.

- Scientific advice or protocol assistance prior to the MAA submission (Yes/No).
- Accelerated Review. Article 14 (9) of Regulation (EC) No 726/2004 (Yes/No).
- Conditional, Exceptional or Standard marketing authorisation granted.
- Orphan at time of CHMP opinion (Yes/No).
- MAH/MAA.
- Date of latest renewal Commission Decision if not full MA or withdrawn at 31 March 2025.
- Current status of CMA at 31 March 2025: conditional MA, converted to full MA, withdrawn MA and the date of the decision.
- Stage of assessment procedure of CMA: During the procedure proposed by applicant, during the procedure proposed by CHMP, during the procedure proposed by CHMP and CAT, initial MAA-requested by applicant.
- Categories of scope for conditional marketing authorisations detailed in EPAR document (Commission Regulation No 507/2006 of 29 March 2006 on the conditional marketing authorisation for medicinal products for human use falling within the scope of Regulation No 726/2004):
    - Art. 2(1): seriously debilitating or life-threatening conditions;
    - Art. 2(2): emergency situations;
    - Art. 2(3): orphan Indication(s) by disease or biomarker (number of indications by disease or biomarker) of each approved drug
- Indication(s) by disease or biomarker of the first approval.
- Specific Obligations imposed to complete post-authorisation measures for the conditional marketing authorisation and due date.

1.2. Methodological variables extracted

- The variables related to the methodology aspects of the main/pivotal studies in the first EPAR published supporting a CMA approval were collected, as well as the data on the results provided by those studies at the time of first authorisation by European Commission. For each pivotal study, the following variables were collected (If more than one pivotal study, all variables were collected separately for each of one):
    - Identification of main study/pivotal study: Sponsor Protocol Code Number.
    - Name of the study.
    - Phase of the studies.
    - Design of main/pivotal study: single-arm, multi-arm non-randomised, multi-arm randomised.
    - Type of control arm in multi-arm main/pivotal studies: Active control=standard of care, different posology of CMA product, not applicable if single-arm study design.
    - Blinding.
    - Types of primary endpoints/outcomes.
    - Result(s) of Primary efficacy endpoint value obtained.
    - Result(s) of Secondary efficacy endpoint value obtained (PFS/OS).
    - Last efficacy analysis done (cut-off date).
    - Intention to treat (ITT) or per protocol (PP) results.
    - N for main variable of EFFICACY used to analyse primary endpoint (including confidence interval).
    - N for main variable of safety (Number of patients with Study Drug Exposure considered for safety analysis in the first EPAR), and if the number of subjects for safety analysis comprise patients from other than pivotal studies (Yes/No).

1.3. ESMO–MCBS assessment

Clinical benefit was assessed using the European Society for Medical Oncology Magnitude of Clinical Benefit Scale (ESMO–MCBS): https://www.esmo.org/guidelines/esmo-mcbs

Version 2.0 was used for solid tumours and version 1.0 for haematological malignancies.

1.4. Clinical practice guideline assessment

Incorporation into clinical practice guidelines was evaluated using the most recent disease-specific European oncology or haematology guideline available as of 31 May 2026.

We recorded the year of publication of the guideline, recommended clinical role, the reported level and/or strength of recommendation, other medicines with same clinical role and same level/strength of recommendation.

1.5. Reimbursement data

Reimbursement status in Spain was obtained from the Spanish Ministry of Health medicines database: https://www.sanidad.gob.es/profesionales/medicamentos.do

Data were accessed on 15 December 2025.

Supplementary Table 1. Primary efficacy outcomes and methodological characteristics of pivotal studies supporting oncology conditional marketing authorisations (CMA) granted between 2017 and 2022

**Active Substance (study identification)**	**Primary outcomes**	**Null (H0) and alternative (Ha) hypotheses tested for primary outcome**	**Sample size analysed for main efficacy variable**	**Result of Primary main outcomes**	**Primary main outcome Confidence Interval**
Alectinib (NP28761)	ORR according to RECIST 1.1 as determined by an IRC	H0: ORR = 35% versus Ha: ORR ≠ 35%	67	ORR = 52.2%	95% CI: 39.7–64.6
Alectinib (NP28673)	Co-Primary efficacy endpoint: 1) ORR as per central IRC using RECIST v1.1 in the overall population (with and without prior exposure of cytotoxic chemotherapy treatments). 2) ORR as per central IRC using RECIST v1.1 in patients with prior exposure of cytotoxic chemotherapy treatments.	H0: ORR=35% versus Ha: ORR ≠ 35%	Co-Primary endpoint: 1) ORR Overall RE Population= 122. 2) ORR Chemotherapy-Pretreated Patients = 96	Co-Primary efficacy endpoint: 1) ORR = 50.8%, 2) ORR = 44.8%	Co-Primary efficacy endpoint: 1) ORR Overall RE Population (95% CI: 41.6–59.9). 2) ORR Chemotherapy-Pretreated (95% CI: 34.6–55.3)
Amivantamab (Protocol number 61186372EDI1001)	ORR as assessed using RECIST v1.1 criteria as evaluated by the investigator. ORR assessed by blinded IRC (BIRC) will also be analysed	For the subgroup of Exon 20 insertion mutant NSCLC (Cohort D) H0: ORR ≤12%. H1 ORR >25%	114	ORR (by investigator assessment) = 36.8%. ORR (BICR assessment) = 43.0%	ORR by investigator assessment (95% CI: 28.0–46.4). ORR by BICR (95% CI: 33.7–52.6)

Avapritinib (BLU-285-1101)	Objective Response Rate (ORR) Determined by Central Radiology Assessment (IRC) according to modified RECIST version 1.1	ORR of 71% (95% CI: 52%–86%)	38 (for Part 2, Cohort 2 of the study)	ORR = 94.7%	95% CI: 82.3–99.4
Avelumab (EMR100070-003)	ORR according to RECIST 1.1 as determined by an IERC	H0: ORR ≤20 versus Ha: ORR >20%	88	ORR = 33%	95% CI: 22.3–43.8
Belantamab Mafodotin (205678)	ORR (defined as sCR+CR+VGPR+PR) according to 2016 International Myeloma Working Group (IMWG) Response Criteria and as assessed by Independent Review Committee (IRC) based on intention to treat (ITT) population.	H0: ORR ≤15%, H1: ORR >15%	N=196. 97 patients (arm with 2.5 mg/kg) + 99 patients (arm with 3.4 mg/kg)	ORR = 33% in the 2.5 mg/kg dose arm, ORR = 32% in the 3.4 mg/kg dose arm	97.5% CI: 22.6–44.7 in the 2.5 mg/kg dose arm, 97.5% CI: 22.1–43.9 in the 3.4 mg/kg dose arm
Brexucabtagene Autoleucel (KTE-C19-102)	ORR according to the 2014 Lugano classification (Cheson et al 2014) as determined by IRC (independent Radiology review committee)	H0: ORR ≤25%, H1: ORR >25%	n=74	ORR = 84%	95% CI (per the Clopper–Pearson method): 73.4–91.3

Cemiplimab (R2810-ONC-1540)	ORR assessed by Independent Review Committee (IRC). Objective response rate was based on a centrally reviewed evaluation at each time point at which a response assessment occurred using RECIST 1.1 or the composite response criteria.	Cohort 1 (mCSCC): H0: ORR =15% vs. H1: ORR ≠15%. Cohort 2 (laCSCC): H0: ORR =25% vs. H1: ORR ≠25%. Cohort 3 (mCSCC): H0: ORR =15% vs. H1: ORR ≠15%	Cohort 1 (59), Cohort 2 (78), Cohort3 (56) =193	Cohort 1 ORR =49.2%, Cohort 2 ORR =43.6%, Cohort 3 ORR =39.3%	Cohort 1 (95% CI: 35.9–62.5), Cohort 2 (95% CI: 32.4–55.3), Cohort 3 (95% CI: 26.5–53.2)
Ciltacabtagene Autoleucel (68284528MMY2001)	The primary efficacy endpoint for the Phase 2 study was planned to be ORR assessed by Independent Review Committee (IRC) according to International IMWG response criteria	H1: ORR >30%	All enrolled 113 (ITT), All treated 97 (mITT)	ITT ORR = 84.1%, mITT ORR = 97.9%	ITT (95% CI: 76.0–90.3), mITT (95% CI: 92.7–99.7)
Dostarlimab (Study 4010-01-001)	1) ORR per RECIST v1.1 by blinded independent central review (BICR), 2) DOR as assessed per RECIST v1.1 based on BICR, or death due to any cause	For Cohort A1: H0 ≤20%, H1 >40%	108	ORR = 43.5%. Median DOR in responders, months (range): not evaluable (At the time of data cut-off 01 March 2020, the median DOR had not been reached and ranged from 2.6 to 28.1+. Probability of maintaining response at 6 months by K–M= 97.9 %)	ORR (95% CI: 34.0–53.4). DOR not evaluable (Probability of maintaining response at 6 months by K–M 95 % CI: 85.8–99.7)
Entrectinib (pooled analysis of three studies: ALKA-372-001 [ALKA], RXDX-101-01 [STARTRK-1], and RXDX-101-02 [STARTRK-2])	1) ORR per RECIST 1.1 by BICR. 2) DOR per RECIST 1.1 by BICR.	NA	N = 74	ORR = 63.5%, DOR=12.9 months	ORR (95% CI: 51.5–74.4). DOR (95% CI: 9.3–NE)

Entrectinib (pooled analysis of three studies: ALKA-372-001 [ALKA], RXDX-101-01 [STARTRK-1], and RXDX-101-02 [STARTRK-2])	1) ORR per RECIST 1.1 by BICR. 2) DOR per RECIST 1.1 by BICR.	NA	N = 94	ORR =73.4%, DOR=16.5 months	ORR (95% CI) = 63.3–82.0. DOR (95% CI) =14.6–28.6
Idecabtagene Vicleucel (BB2121-MM-001)	ORR assessed by Independent Review Committee (IRC) according to International IMWG response criteria (Kumar, 2016)	H0: ORR ≤50%. H1: >50%, with a target ORR of 70%.	n=140	ORR = 67.1%	95% CI (Clopper–Pearson exact): 59.4–74.9
Larotrectinib (pooled analysis of three studies: LOXO-TRK-14001, LOXO-TRK-15002 [NAVIGATE], and LOXO-TRK-15003	ORR according to RECIST 1.1 as determined by an IRC	H0: ORR ≤30%, H1: ORR >50%	N = 73	ORR = 75%	95% CI = 64–85
Loncastuximab Tesirine (ADCT-402-201)	ORR according to the 2014 Lugano classification (Cheson et al 2014) as determined by central review in all-treated patients. ORR was defined as the proportion of patients with a best overall response of CR or PR.	H0: ORR ≤0.2% vs. H1: ORR >0.2%	145	ORR = 48.3%	95% CI: 39.9–56.7
Lorlatinib (B7461001)	1) ORR according to RECIST 1.1. as determined by an IRC. 2) Intracranial ORR (IC ORR) according to RECIST 1.1. as determined by an IRC	Exploratory: Non-comparative study. There was no sample size definition based on either expected desirable	Patients with ALK-positive NSCLC were included in various Cohorts according to prior treatment. 1) Cohort 2–3A (N=59), 2) Cohort 3B	1) Cohort 3B, ORR = 42.9%. 2) Cohort 4 –5, ORR = 38.7%. 3) Cohort 2– 3A, ORR = 69.5%	1) Cohort 3B (95% CI: 24.5–62.8). 2) Cohort 4–5 (95% CI: 29.6–48.5). 3) Cohort 2–3A (95% CI: 56.1–80.8)

		outcomes or a prespecified precision of the estimates for the hypothesised ORR, as demonstrated by the large width of the 95% CI listed in the table	(N=28), 3) Cohort 4–5 (N=111. Total N=198		
Mosunetuzumab (GO29781)	CR rate by Independent Review Facility (IRF) (Complete Remission rate assessed by Independent Review Committee) using according to standard non-Hodgkin's lymphoma (NHL) response criteria (Cheson et al. 2007). CR rate is defined as the proportion of patients whose best overall response is a CR based upon IRF assessment	H0: CR <14%. H1 CR ≥14% (For Cohort B11 RP2D, the expansion Cohorts for FL have been designed to rule out a 14% CR rate and are powered to detect a 14% increase in CR rate from 14% to 28%)	90	Cohort B11 FL RP2D the CR, by IRF, CR=60%	Cohort B11 (95% CI: 49.1–70.2)
Pemigatinib (INCB54828-202)	ORR in participants with FGFR2 rearrangements or fusions based on the central genomics laboratory results (Cohort A) based on RECIST v1.1 assessed by IRC.	H1 >15%	108	ORR = 37.0%	95% CI: 27.94–46.86
Polatuzumab Vedotin (GO29365)	Complete Response (CR) as measured by PET–CT scan as determined by an IRC assessed	Exploratory: An exploratory comparison of CR rates for the BR-containing regimens was conducted using the Cochran Mantel Haenszel chi–square test adjusted for randomization stratification factors.	N=80. 40 patients Arm C Pola+BR) + 40 patients Arm D (BR)	Arm C Pola+BR, CR=40.0%. Arm D BR, CR=17.5%	Arm C Pola+BR (95% CI: 24.9–56.7). Arm D BR (95% CI: 7.3–32.8). P = 0.0261

Pralsetinib (BLU-667-1101)	ORR per RECIST v1.1 by blinded independent central review (BICR)	Cohort 1, H0: ORR = 0.23% versus the H1 ORR = 0.5%. Cohort 2, H0: ORR = 0.48% versus the H1 ORR = 0.61%	Cohort 1, N= 136. Cohort 2, N=75. Overall =233 (including Cohort 1, 2 and Prior non-platinum systemic treatment)	Cohort 1, ORR =58.8%. Cohort 2 (No prior systemic treatment), ORR = 72.0%. Overall, ORR = 64.4%	Cohort 1 (95% CI: 50.1–67,2). Cohort 2 (95% CI: 60.4–81.8). Overall (95% CI: 57.9–70.5).
Rucaparib (pooled analysis of two studies: CO-338-010 Part 2A and CO-338-017 Parts 1 and 2)	ORR by investigator (RECIST 1.1)	NA	106 (Study CO-338-010 Part 2A gBRCA, N = 42. Study CO-338-017 Part 1 BRCA subgroup, N = 24. Study CO-338-017 Part 2 BRCA subgroup, N = 40 patients)	ORR = 54.7%	95% CI = 44.8–64.4
Selinexor (Study KCP-330-012, Part 2)	ORR assessed by Independent Review Committee (IRC) according to International IMWG response criteria (Kumar, 2016)	H0 ≤0.10%, H1 >0.20%	83	ORR (BCLPD-refractory) =25.3%	95% CI: 16.4–36
Selpercatinib (LOXO-RET-17001)	ORR according to RECIST 1.1 as determined by an IERC (for each Phase 2 expansion Cohort)	Cohort NSCLC. H0: ORR< 50%, H1: ORR of ≥50%. Cohort MTC. H0: ORR <40%, H1: ORR ≥40%. Cohort TC. No formal hypothesis was specified for RET-fusion positive TC.	Cohort NSCLC: for PAS N= 105, for IAS N=218, for SAS1 N= 48. Cohort MTC: for PAS N= 55, for IAS N=143, for SAS1 N= 90. Cohort TC: for PAS N=19	Cohort NSCLC: for PAS ORR = 63.8, for IAS ORR = 56.9, for SAS1 ORR =85.4. Cohort MTC: for PAS ORR = 69.1%, for IAS ORR = 69.2%, for SAS1 ORR=76.7%. Cohort TC: for PAS ORR = 78.9%	Cohort NSCLC: for PAS 95% CI=53.9–73.0, for IAS 95% CI=50.0–63.6, for SAS1 95% CI= 72.2–93.9. Cohort MTC: for PAS 95% CI= 55.2–80.9, for IAS 95% CI=61.0–76.7, for SAS1 95% CI= 66.6–84.9.

					Cohort TC: for PAS 95% CI=54.4–93.9
Selumetinib (D1532C00057)	ORR (Responses were assessed by NCI POB central analysis using 3D-volumetric MRI according to REiNS recommendations for imaging tumour response in neurofibromatosis clinical trials. Assessment was performed by a single reader which is considered subjective)	ORR H0 ≤15%, H1 >36%	50	ORR = 66%	95% CI: 51.2–78.8%
Sotorasib (20170543)	ORR assessed per RECIST 1.1 by blinded independent central review (BICR)	ORR H0 ≤32%, H1 >32% (subjects with NSCLC)	124	ORR = 37.1%	95% CI: 28.6–46.2
Tafasitamab (MOR208C203)	ORR as assessed by Independent Radiology/Clinical Review Committee (IRC). Disease response assessments were made according to the revised response criteria based on the guidelines of the International Working Group (IWG) reported by Cheson et al. 2007.	ORR >20% (under monotherapy), ORR >35% (under combination therapy: Tafasitamab in combination with lenalidomide)	81	ORR (Tafasitamab+Lenalidomide) = 56.8%	95% CI: 45.3–67.8

Teclistamab (64007957MMY1001; Phase 2 CR108859 study)	ORR assessed by Independent Review Committee (IRC) according to International IMWG response criteria	Cohort A, the H1 is ORR >45%. Cohort C the H1 is ORR >35%	Cohort A: 165. Cohort C: 40	Cohort A, ORR = 63.0%. Cohort C, ORR = 52.5%	Cohort A, 95% CI: 55.2–70.4. Cohort C, 95% CI: 36.1–68.5
Trastuzumab Deruxtecan (pooled analysis of two studies: DS8201-A-J101 [J101] and DS8201-A-U201)	ORR according to RECIST 1.1 as determined by an IRC	NA	N = 235	ORR = 58.3 %	95% CI = 51.7–64.7

Note: Alectinib and Entrectinib entry appears duplicated because two studies were included

Abbreviations: BICR, blinded independent central review; BR, bendamustine plus rituximab; CI, confidence interval; CMA, conditional marketing authorisation; CR, complete response; DOR, duration of response; FL, follicular lymphoma; IAS, integrated analysis set; IERC, independent endpoint review committee; IMWG, International Myeloma Working Group; IRC, independent review committee; IRF, independent review facility; ITT, intention-to-treat; K–M, Kaplan–Meier; mITT, modified intention-to-treat; MRI, magnetic resonance imaging; MTC, medullary thyroid cancer; NA, not applicable; NHL, non-Hodgkin lymphoma; NSCLC, non-small cell lung cancer; ORR, overall response rate; OS, overall survival; PAS, primary analysis set; PET–CT, positron emission tomography–computed tomography; PFS, progression-free survival; PP, per protocol; PR, partial response; RECIST, Response Evaluation Criteria in Solid Tumours; REiNS, Response Evaluation in Neurofibromatosis and Schwannomatosis; RP2D, recommended Phase 2 dose; SAS, safety analysis set; TC, thyroid cancer; VGPR, very good partial response.

Supplementary Table 2. Secondary efficacy outcomes reported in pivotal studies supporting oncology conditional marketing authorisations (CMA) granted between 2017 and 2022.

Active Substance (study identification)	Median PFS (months)	PFS hazard ratio (HR, 95% CI) in multi-arm studies	% PFS	PFS largest time obtained (months)	Median OS (months)	Hazard rate of OS. If multi-arm design = HR (95% CI)	% OS	OS largest time obtained (months)
Alectinib (NP28761)	8.2	NA	Not reported	Not reported	22.7	NA	Not reported	Not reported
Alectinib (NP28673)	8.9 months	NA	Not reported	Not reported	26.0 months	NA	Not reported	Not reported
Amivantamab (Protocol number 61186372EDI1001)	6.93 (by Investigator)	NA	18 (by Investigator)	24 (by Investigator)	22.77	NA	40	30
Avapritinib (BLU-285-1101)	24	NA	NA	NA	Not reached	NA	70.60%	36
Avelumab (EMR100070-003)	2.7	NA	30	15	12.9	NA	44	15
Belantamab Mafodotin (205678)	Cohort 2.5 mg/kg: 2.8; Cohort 3.4 mg/kg: 3.9	0.96 (95% CI: 0.69–1.35)	NA	NA	13.7 months in the 2.5 mg/kg Cohort. Not reported in the 3.4 mg/kg Cohort	NA	Cohort 2.5 mg/kg: 72%; Cohort 3.4 mg/kg: 75%	6

Brexucabtagene Autoleucel (KTE-C19-102)	16.2	NA	43	36	Not reached	NA	63.2	36
Cemiplimab (R2810-ONC-1540)	Total: 18.4 (Cohort 1: 18.4; Cohort 2: Not reported, Cohort 3: 10.4)	NA	Total: 51 (Cohort 1: 53.1; Cohort 2: 51.8, Cohort 3: NE)	16	Not reached	NA	Total: 85.7% (Cohort 1: 81.3; Cohort 2: 93.2, Cohort 3: 76.1)	12
Ciltacabtagene Autoleucel (68284528MMY2001)	24.31	NA	70.5	12	Not reached	NA	81.5	12
Dostarlimab (Study 4010-01-001)	5.5	NA	46.9	12	Not reached	NA	68.8	12
Entrectinib (pooled analysis of three studies: ALKA-372-001 [ALKA], RXDX-101-01 [STARTRK-1], and RXDX-101-02 [STARTRK-2])	19	NA	52	18	Not reached	NA	82	18
Entrectinib (pooled analysis of three studies: ALKA-372-001 [ALKA], RXDX-101-01 [STARTRK-1], and RXDX-101-02 [STARTRK-2])	11.2	NA	Not estimated	Not estimated	23.9	NA	Not estimated	Not estimated
Idecabtagene Vicleucel (BB2121-MM-001)	8.3	NA	20.2	21	21.4	NA	51.9	21

Larotrectinib (pooled analysis of three studies: LOXO-TRK-14001, LOXO-TRK-15002 [NAVIGATE], and LOXO-TRK-15003	27.4	NA	64	12	Not reached	NA	88	12
Loncastuximab Tesirine (ADCT-402-201)	4.93	NA	Not reported	Not reported	9.53	NA	Not reported	Not reported
Lorlatinib (B7461001)	Cohort 3B: 5.5; Cohort 4–5: 6.9	NA	Cohort 3B: 21.9%; Cohort 4–5: 23.1%	18	Cohort 3B: 21.1; Cohort 4–5: 19.2	NA	Cohort 3B: 61.6%; Cohort 4–5: 54.2%	18
Mosunetuzumab (GO29781)	17.9	NA	57.7	12	Not reached	NA	93	12
Pemigatinib (INCB54828-202)	7.03 (Cohort A)	NA	Cohort A: 29.2. Cohort B and Cohort C not reached	12 (Cohort A)	17.48 (Cohort A)	NA	76.1	12
Polatuzumab Vedotin (GO29365)	Treatment arm 9.5; control arm 3.7	0.36 (0.21, 0.63); p = 0.0002	NA	NA	Treatment arm 12.4; control arm 4.7	0.42 (0.24, 0.75); p = 0.0023	NA	NA
Pralsetinib (BLU-667-1101)	16.4	NA	42.1	24	Not reached	NA	66	24
Rucaparib (pooled analysis of two studies: CO-338-010 Part 2A and CO-338-017 Parts 1 and 2)	9.55	NA	Not reported	Not reported	Not evaluated	NA	Not evaluated	Not evaluated

Selinexor (Study KCP-330-012, Part 2)	3.7	NA	Not reported	Not reported	8.4	NA	37.7	12
Selpercatinib (LOXO-RET-17001)	Cohort NSCLC: 19.29; Cohort MTC: not reached; Cohort TC: 20.07	NA	Cohort NSCLC: 43.7%; Cohort MTC: 61.4%; Cohort TC: 44.9	Cohort NSCLC: 24; Cohort MTC: 24; Cohort TC: 24	Cohort NSCLC: not reached; Cohort MTC: 33.25; Cohort TC: not evaluated	NA	Cohort NSCLC: 67.3%; Cohort MTC: 76.7%; Cohort TC: not evaluated	Cohort NSCLC: 24 months; Cohort MTC: 24 months; Cohort TC: not evaluated
Selumetinib (D1532C00057)	Not reached	NA	Not reported	NA	Not assessed	NA	NA	NA
Sotorasib (20170543)	6.8	NA	16.3	12	12.5	NA	51.4	12
Tafasitamab (MOR208C203)	12.1	NA	41.5	30	31.6	NA	46.1	36
Teclistamab (64007957MMY1001; Phase 2 CR108859 study)	Cohort A: 10.1 (cohort C not evaluated outcome)	NA	48.3	12	Cohort A: 18.3. (Cohort C not evaluated outcome)	NA	77.2	9
Trastuzumab Deruxtecan (pooled analysis of two studies: DS8201-A-J101 [J101] and DS8201-A-U201)	13.9	NA	73.6	Not reported	Not evaluated	Not evaluated	Not evaluated	Not evaluated

Abbreviations: CI, confidence interval; CMA, conditional marketing authorisation; HR, hazard ratio; NA, not applicable; NE, not estimable; NSCLC, non-small cell lung cancer; OS, overall survival; PFS, progression-free survival; TC, thyroid cancer; MTC, medullary thyroid cancer.

Supplementary Table 3. Sample size used for safety analyses in pivotal studies supporting oncology conditional marketing authorisations (CMA) granted between 2017 and 2022.

Active Substance (study identification)	Sample size analysed for main efficacy variable	Safety analysis sample size
Alectinib (NP28761)	67	253
Alectinib (NP28673)	Co-Primary endpoint: 122 and 96	
Amivantamab (Protocol number 61186372EDI1001)	114	489
Avapritinib (BLU-285-1101)	38	585
Avelumab (EMR100070-003)	88	1738
Belantamab Mafodotin (205678)	196	291
Brexucabtagene Autoleucel (KTE-C19-102)	74	82
Cemiplimab (R2810-ONC-1540)	193	591
Ciltacabtagene Autoleucel (68284528MMY2001)	97	106
Dostarlimab (Study 4010-01-001)	108	515
Entrectinib (pooled analysis of three studies: ALKA-372-001 [ALKA], RXDX-101-01 [STARTRK-1], and RXDX-101-02 [STARTRK-2])	74	504
Entrectinib (pooled analysis of three studies: ALKA-372-001 [ALKA], RXDX-101-01 [STARTRK-1], and RXDX-101-02 [STARTRK-2])	94	
Idecabtagene Vicleucel (BB2121-MM-001)	140	184
Larotrectinib (pooled analysis of three studies: LOXO-TRK-14001, LOXO-TRK-15002 [NAVIGATE], and LOXO-TRK-15003	73	176
Loncastuximab Tesirine (ADCT-402-201)	145	215
Lorlatinib (B7461001)	198	332
Mosunetuzumab (GO29781)	90	414
Pemigatinib (INCB54828-202)	108	562
Polatuzumab Vedotin (GO29365)	80	588
Pralsetinib (BLU-667-1101)	233	528
Rucaparib (pooled analysis of two studies: CO-338-010 Part 2A and CO-338-017 Parts 1 and 2)	106	409
Selinexor (Study KCP-330-012, Part 2)	83	214
Selpercatinib (LOXO-RET-17001)	Cohort NSCLC: 105. Cohort MTC: 55. Cohort TC: 19	746
Selumetinib (D1532C00057)	50	347
Sotorasib (20170543)	124	456
Tafasitamab (MOR208C203)	81	222

Teclistamab (64007957MMY1001; Phase 2 CR108859 study)	Cohort A: 165. Cohort C: 40	342
Trastuzumab Deruxtecan (pooled analysis of two studies: DS8201-A-J101 [J101] and DS8201-A-U201)	N = 235	542

For Brexucabtagene autoleucel and Ciltacabtagene autoleucel, the safety population corresponded exclusively to patients included in the pivotal efficacy study. For all other medicines, the safety population included patients from additional supportive studies.

Abbreviations: CMA, conditional marketing authorisation.

Supplementary Table 4. Regulatory characteristics, pivotal study design, and efficacy outcomes of oncology medicines granted conditional marketing authorisation (CMA) between 2017 and 2022

Active substance	WHO ATC 3rd level, last updated on 26 January 2024,	Date of 1st authorisation	Specific obligations (n)	Date of final outcome (SMA or withdrawal)	Regulatory status at 31/03/2025	Number of pivotal studies	Phase of pivotal study	Design of pivotal study	Primary main outcomes	Median PFS (months)	Median OS (median time in months for total data. If multi-arm, data of treatment arm)	Sample size analysed for the primary efficacy endpoint
Alectinib	Protein kinase inhibitors	16/02/2017	1	01/12/2017	SMA	2	II (both)	Single-arm (both)	ORR (both pivotal studies)	Study 1: 8.2, Study: 2: 8.9	Study 1: 22.7, Study 2: 26.0	Study 1: 67. Study 2: Co-Primary endpoint with 122 and 96
Amivantamab	Monoclonal antibodies and antibody drug conjugates	09/12/2021	1	27/06/2024	SMA	1	I (Part 2)	Single-arm	ORR	6.93 (by Investigator)	22.77	114
Avapritinib	Protein kinase inhibitors	24/09/2020	3	NA	CMA	1	I (Part 2)	Single-arm	ORR	24	Not reached	38
Avelumab	Monoclonal antibodies and antibody drug conjugates	18/09/2017	1	19/08/2020	SMA	1	II	Single-arm	ORR	2.7	12.9	88
Belantamab Mafodotin	Monoclonal antibodies and antibody drug conjugates	25/08/2020	2	14/12/2023	Withdrawn MA	1	II	Multi-arm randomised (Parallel Assignment)	ORR	Cohort 2.5 mg/kg: 2.8; Cohort 3.4 mg/kg: 3.9	13.7 months in the 2.5 mg/kg Cohort. Not reported in the 3.4 mg/kg Cohort	196
Brexucabtagene Autoleucel	Other antineoplastic agents	14/12/2020	2	NA	CMA	1	II	Single-arm	ORR	16.2	Not reached	74
Cemiplimab	Monoclonal antibodies and antibody drug conjugates	28/06/2019	2	01/07/2022	SMA	1	II	Single-arm	ORR	Total: 18.4 (Cohort 1: 18.4; Cohort 2: not	Not reached	193

										reported, Cohort 3: 10.4)		
Ciltacabtagene Autoleucel	Other antineoplastic agents	25/05/2022	2	19/04/2024	SMA	1	II	Single-arm	ORR	24.31	Not reached	113
Dostarlimab	Monoclonal antibodies and antibody drug conjugates	21/04/2021	2	07/12/2023	SMA	1	I (Part 2B)	Single-arm	ORR	5.5	Not reached	108
Entrectinib	Protein kinase inhibitors	31/07/2020	2	NA	CMA	2	Pooled analysis of 3 studies (2 Phase I + 1 Phase II)	Single-arm (both)	ORR and DOR (both pivotal studies)	Study 1: 19, Study 2: 11.2	Study 1: not reached, Study 2: 23.9	Study 1 with 74 and study 2 with 94
Idecabtagene Vicleucel	Other antineoplastic agents	18/08/2021	2	19/03/2024	SMA	1	II	Single-arm	ORR	8.3	21.4	140
Larotrectinib	Protein kinase inhibitors	19/09/2019	3	NA	CMA	1	Pooled analysis of 3 studies (1 Phase I + 1 Phase I/II + 1 Phase II)	Single-arm	ORR	27.4	Not reached	73
Loncastuximab Tesirine	Monoclonal antibodies and antibody drug conjugates	20/12/2022	2	NA	CMA	1	II	Single-arm	ORR	4.93	9.53	145
Lorlatinib	Protein kinase inhibitors	06/05/2019	2	NA	CMA	1	II	Single-arm	ORR	(Cohort 3B: 5.5; Cohort 4-5: 6.9)	(Cohort 3B: 21.1; Cohort 4-5: 19.2)	198
Mosunetuzumab	Monoclonal antibodies and antibody drug conjugates	03/06/2022	1	NA	CMA	1	II	Single-arm	CR	17.9	Not reached	90

Pemigatinib	Protein kinase inhibitors	26/03/2021	2	NA	CMA	1	II	Single-arm	ORR	7.03 (Cohort A)	17.48 (Cohort A)	108
Polatuzumab Vedotin	Monoclonal antibodies and antibody drug conjugates	16/01/2020	2	24/05/2022	SMA	1	II	Multi-arm randomised (Parallel Assignment)	CR	Treatment arm 9.5; control arm 3.7	Treatment arm 12.4; control arm 4.7	80
Pralsetinib	Protein kinase inhibitors	18/11/2021	2	24/10/2024	Withdrawn MA	1	II	Single-arm	ORR	16.4	Not reached	233
Rucaparib	Other antineoplastic agents	24/05/2018	1	09/11/2022	SMA	1	Pooled analysis of 2 studies (2 Phase II)	Single-arm	ORR	9.55	Not evaluated	106
Selinexor	Other antineoplastic agents	26/03/2021	1	18/07/2022	SMA	1	IIb (Part 2)	Single-arm	ORR	3.7	8.4	83
Selpercatinib	Protein kinase inhibitors	11/02/2021	3	NA	CMA	1	II	Single-arm	ORR	Cohort NSCLC: 19.29; Cohort MTC: not reached; Cohort TC: 20.07	Cohort NSCLC: not reached; Cohort MTC: 33.25; Cohort TC: not evaluated	Cohort NSCLC with 105, Cohort MTC with 55, Cohort TC with 19
Selumetinib	Protein kinase inhibitors	17/06/2021	3	NA	CMA	1	II	Single-arm	ORR	Not reached	Not assessed	50
Sotorasib	Other antineoplastic agents	06/01/2022	1	NA	CMA	1	II	Single-arm	ORR	6.8	12.5	124
Tafasitamab	Monoclonal antibodies and antibody drug conjugates	26/08/2021	3	NA	CMA	1	II	Single-arm	ORR	12.1	31.6	81
Teclistamab	Monoclonal antibodies and antibody drug conjugates	23/08/2022	2	NA	CMA	1	II (Part 3)	Single-arm	ORR	Cohort A: 10.1 (Cohort C not evaluated outcome)	Cohort A: 18.3. (Cohort C not evaluated outcome)	Cohort A with 165, Cohort C with 40
Trastuzumab Deruxtecan	Monoclonal antibodies and antibody drug conjugates	18/01/2021	1	NA	CMA	1	Pooled analysis of 2 studies (1	Single-arm	ORR	13.9	Not evaluated	235

							Part 2 of Phase I + 1 Phase II)					

Belantamab Mafodotin underwent non-renewal of its conditional marketing authorisation following an EMA recommendation, whereas Pralsetinib was withdrawn at the request of the marketing authorisation holder.

Abbreviations: ATC, Anatomical Therapeutic Chemical; CI, confidence interval; CMA, conditional marketing authorisation; CR, complete response; DOR, duration of response; HR, hazard ratio; MA, marketing authorisation; MTC, medullary thyroid cancer; NA, not applicable; NSCLC, non-small cell lung cancer; ORR, overall response rate; OS, overall survival; PFS, progression-free survival; SMA, standard marketing authorisation; SO, specific obligation; TC, thyroid cancer.

Supplementary Table 5. Multivariable logistic regression analysis of factors associated with conversion from conditional marketing authorisation (CMA) to standard marketing authorisation (SMA).

**Characteristic**	**OR**	**95% CI**	**P-value**
Group			
2006–2016	—	—	
2017–2022	2.14	0.35–19.1	0.4
Pivotal study Phase			
Phase 1	—	—	
Phase 2	0.48	0.05–4.86	0.5
Phase 3	1.69	0.05–83.2	0.8
Study design			
Single-arm	—	—	
Multi-arm	1.39	0.14–11.6	0.8
Primary efficacy sample size	1.00	0.99–1.00	0.8

Both multi-arm studies were phase II trials; no phase III multi-arm pivotal studies were identified. Owing to the limited number of events and the strong imbalance across study design and phase categories, regression analyses should be interpreted as exploratory. P values were not adjusted for multiple comparisons.

Abbreviations: CI, confidence interval; CMA, conditional marketing authorisation; OR, odds ratio; SMA, standard marketing authorisation.

Supplementary Table 6. Incorporation of oncology medicines granted conditional marketing authorisation (CMA) between 2017 and 2022 into ESMO and EHA clinical practice guidelines.

Active substance	First CMA approved indication	Regulatory status (31 Mar 2025)	ESMO guideline	Recommended clinical role	Level/strength of recommendation	Other medicines with same clinical role and same level/strength of recommendation	Other medicines	Level/strength of recommendation	Guideline year
Alectinib	ALK-positive advanced NSCLC	SMA	Pan-Asian adapted ESMO Clinical Practice Guidelines for the diagnosis, treatment and follow-up of patients with oncogene-addicted metastatic non-small-cell lung cancer	Preferred targeted therapy	IA	YES	Patients should be treated in the first-line setting with Alectinib, Brigatinib, Ensartinib or Lorlatinib	IA	2024
Amivantamab	Advanced NSCLC with activating EGFR Exon 20 insertion mutations, after chemotherapy failure	SMA	Pan-Asian adapted ESMO Clinical Practice Guidelines for the diagnosis, treatment and follow-up of patients with oncogene-addicted metastatic non-small-cell lung cancer	Preferred targeted therapy	IIB	NO	Amivantamab monotherapy is an option after platinum-based chemotherapy failure for the treatment of EGFR exon insertion-mutated NSCLC not previously exposed to Amivantamab	IIB	2024
Avapritinib	Unresectable or metastatic GIST harbouring the PDGFRA D842V mutation	CMA	Gastrointestinal stromal tumours: ESMO-EURACAN-GENTURIS Clinical Practice Guidelines for diagnosis, treatment and follow-up	Preferred first-line therapy	IIIA	NO	NA	NA	2021
Avelumab	Metastatic Merkel cell carcinoma	SMA	Merkel-cell carcinoma: ESMO–EURACAN Clinical Practice Guideline for diagnosis, treatment and follow-up	First and second-line therapy	IIIA	YES	Pembrolizumab, Retifanlimab, Nivolumab	IIIA	2024

Belantamab Mafodotin	Multiple myeloma progression after four prior therapies	Withdrawn MA	Withdrawn MA	Withdrawn MA	Withdrawn MA				Withdrawn MA
Brexucabtagene Autoleucel	Relapsed or refractory MCL after two or more lines of systemic therapy including a BTK inhibitor	CMA	Eyre T, Cwynarski K, d'Amore F et al. Lymphomas: ESMO Clinical Practice Guideline for diagnosis, treatment and follow-up. Annals of Oncology, 2025; 36, 1263-1284	CAR-T cell therapy	IIIA	YES	Pirtobrutinib	IIIA	2025
Cemiplimab	Metastatic or locally advanced cutaneous SCC who are not candidates for curative surgery or curative radiation.	SMA	European consensus-based interdisciplinary guideline for invasive cutaneous squamous cell carcinoma: Part 2. Treatment–Update 2023	Preferred targeted therapy	Strong in favour; low quality of evidence	NO	NA	NA	2023
Ciltacabtagene Autoleucel	Relapsed and refractory multiple myeloma, progression after three prior therapies	SMA	EHA–EMN Evidence-Based Guidelines for diagnosis, treatment and follow-up of patients with multiple myeloma	Later-line therapy	IA	YES	Ciltacabtagene Autoleucel, Idecabtagene Vicleucel, Belantamab Mafodotin, Daratumumab, Isatuximab, Elotuzumab, Carfilzomib, Selinexor	IA	2025
Dostarlimab	dMMR/MSI-H recurrent or advanced endometrial cancer that has progressed	SMA	Endometrial cancer: ESMO Clinical Practice Guideline for diagnosis, treatment and follow-up	Second-line therapy	IIIB	YES	Avelumab, Durvalumab and Dostarlimab	IIIB	2022
Entrectinib	1) Locally advanced, metastatic or where surgical resection is likely to result in severe morbidity solid tumours that have a NTRK gene fusion.	CMA	The ESMO Tumour-Agnostic Classifier and Screener (ETAC-S): a tool for assessing tumour-agnostic potential of molecularly guided therapies and for steering drug development	Tumour-agnostic therapy	NA	NA	NA	NA	2024
Entrectinib	2) ROS1-positive advanced NSCLC not previously treated with ROS1 inhibitors.	CMA	Pan-Asian adapted ESMO Clinical Practice Guidelines for the diagnosis, treatment and follow-up of patients with oncogene-addicted metastatic non-small-cell lung cancer	Preferred targeted therapy	IIIA	YES	Crizotinib or Entrectinib is recommended in the first-line setting	IIIA	2024

Idecabtagene Vicleucel	Relapsed and refractory multiple myeloma, progression after three prior therapies	SMA	EHA–EMN Evidence-Based Guidelines for diagnosis, treatment and follow-up of patients with multiple myeloma	Later-line therapy	IA	YES	Ciltacabtagene Autoleucel, Idecabtagene Vicleucel, Belantamab Mafodotin, Daratumumab, Isatuximab, Elotuzumab, Carfilzomib, Selinexor	IA	2025
Larotrectinib	Locally advanced, metastatic or where surgical resection is likely to result in severe morbidity solid tumours that display a NTRK gene fusion	CMA	The ESMO Tumour-Agnostic Classifier and Screener (ETAC-S): a tool for assessing tumour-agnostic potential of molecularly guided therapies and for steering drug development	Tumour-agnostic therapy	NA	NA	NA	NA	2024
Loncastuximab Tesirine	Relapsed/refractory DLBCL and HGBL after ≥2 prior lines of systemic therapy	CMA	Eyre T, Cwynarski K, d'Amore F et al. Lymphomas: ESMO Clinical Practice Guideline for diagnosis, treatment and follow-up. Annals of Oncology, 2025; 36, 1263-1284	Later-line therapy	IIIB	YES	Loncastuximab Tesirine, Tafasitamab–Lenalidomide	IIIB	2025
Lorlatinib	ALK-positive advanced NSCLC whose disease has progressed after: alectinib, ceritinib or crizotinib	CMA	Pan-Asian adapted ESMO Clinical Practice Guidelines for the diagnosis, treatment and follow-up of patients with oncogene-addicted metastatic non-small-cell lung cancer	Preferred targeted therapy	IA	YES	Patients should be treated in the first-line setting with Alectinib, Brigatinib, Ensartinib or Lorlatinib	IA	2024
Mosunetuzumab	Relapsed or refractory follicular lymphoma who have received at least two prior systemic therapies	CMA	Not included in ESMO guidelines	Not included in ESMO guidelines	NA				NA
Pemigatinib	Locally advanced or metastatic Cholangiocarcinoma with a FGFR2 fusion or rearrangement that have progressed after at least one prior line of systemic therapy	CMA	ESMO Clinical Practice Guideline interim update on the management of biliary tract cancer	Second or later-line therapy	IIIA	YES	Futibatinib	IIIA	2024

Polatuzumab Vedotin	Relapsed/refractory DLBCL not eligible for haematopoietic stem-cell transplant	SMA	Eyre T, Cwynarski K, d'Amore F et al. Lymphomas: ESMO Clinical Practice Guideline for diagnosis, treatment and follow-up. Annals of Oncology, 2025; 36, 1263-1284	Second-line therapy	IIB	NO	NA	NA	2025
Pralsetinib	RET fusion-positive advanced NSCLC not previously treated with a RET inhibitor	Withdrawn MA	Withdrawn MA	Withdrawn MA	Withdrawn MA				Withdrawn MA
Rucaparib	Platinum sensitive, relapsed or progressive, BRCA mutated, high-grade epithelial ovarian, fallopian tube, or primary peritoneal cancer, who have been treated with two or more prior lines of platinum-based chemotherapy	SMA	ESMO Clinical Practice Guideline Express Update on the management of epithelial ovarian cancer	No current ESMO recommendation corresponding to the original CMA treatment indication	NA	NA	NA	NA	2026
Selinexor	Progression of multiple myeloma after four prior therapies	SMA	EHA–EMN Evidence-Based Guidelines for diagnosis, treatment and follow-up of patients with multiple myeloma	Later-line therapy	IA	YES	Ciltacabtagene Autoleucel, Idecabtagene Vicleucel, Belantamab Mafodotin, Daratumumab, Isatuximab, Elotuzumab, Carfilzomib, Selinexor		2025

Selpercatinib	1) Advanced RET-fusion positive NSCLC and advanced RET-fusion positive thyroid cancer following prior treatment with Sorafenib and/or Lenvatinib.	CMA	Pan-Asian adapted ESMO Clinical Practice Guidelines for the diagnosis, treatment and follow-up of patients with oncogene-addicted metastatic non-small-cell lung cancer	Preferred targeted therapy	IA	NO	Treatment with Selpercatinib is recommended as first-line therapy for patients with RET fusion-positive NSCLC	IA	2024
Selpercatinib	2) Adults and adolescents ≥ 12 years with advanced RET mutant MTC following prior treatment with Cabozantinib and/or Vandetanib	CMA	ESMO Clinical Practice Guideline update on the use of systemic therapy in advanced thyroid cancer	Second-line therapy	VB	YES	Pralsetinib but after withdrawn	VB	2022
Selumetinib	Symptomatic, inoperable plexiform neurofibromas in paediatric patients with NF1 aged ≥ 3 years	CMA	NA*	NA*	NA*	NA*	NA*	NA*	NA*
Sotorasib	Advanced NSCLC with KRAS G12C mutation and who have progressed	CMA	Pan-Asian adapted ESMO Clinical Practice Guidelines for the diagnosis, treatment and follow-up of patients with oncogene-addicted metastatic non-small-cell lung cancer	Second-line therapy	IB	YES	Adagrasib is recommended for the treatment of KRAS G12C-mutated NSCLC failing prior therapy	IIIB	2024
Tafasitamab	Relapsed/refractory DLBCL not eligible for autologous stem cell transplant	CMA	Eyre T, Cwynarski K, d'Amore F et al. Lymphomas: ESMO Clinical Practice Guideline for diagnosis, treatment and follow-up. Annals of Oncology, 2025; 36, 1263-1284	Later-line therapy	IIIB	YES	Loncastuximab Tesirine, Tafasitamab–Lenalidomide	IIIB	2025
Teclistamab	Progression of Relapsed and refractory multiple	CMA	EHA–EMN Evidence-Based Guidelines for diagnosis, treatment and follow-up of patients with multiple myeloma	Later-line therapy	IIB	YES	Teclistamab, Elranatamab,	IIB	2025

	myeloma after three prior therapies						Linvoseltamab, Talquetamab		
Trastuzumab Deruxtecan	Unresectable or metastatic HER2-positive breast cancer who have received two or more prior anti HER2 based regimens	CMA	ESMO Clinical Practice Guideline for the diagnosis, staging and treatment of patients with metastatic breast cancer	Second-line therapy	IA	YES	Tucatinib–trastuzumab–capecitabine	IA	2021

*Line-of-therapy classifications were not applicable for selumetinib because ESMO recommendations are based on neurofibromatosis-associated plexiform neurofibromas rather than a sequential oncology treatment pathway.

For each medicine, the first CMA-approved indication was matched to the most recent disease-specific ESMO or EHA guideline available as of 31 May 2026. The table summarises the recommended clinical role, level/strength of recommendation, and whether alternative therapies with the same clinical role and the same level/strength of recommendation were identified. Withdrawn medicines were classified according to their regulatory status as of 31 March 2025. For medicines with multiple CMA-approved indications, each indication was evaluated separately. Recommendations are reported according to the grading systems used in the corresponding guideline.

Abbreviations: CMA, conditional marketing authorisation; SMA, standard marketing authorisation; ESMO, European Society for Medical Oncology; EHA, European Haematology Association; NSCLC, non-small-cell lung cancer; DLBCL, diffuse large B-cell lymphoma; HGBL, high-grade B-cell lymphoma; MCL, mantle cell lymphoma; MTC, medullary thyroid cancer; GIST, gastrointestinal stromal tumour; FGFR2, fibroblast growth factor receptor 2; PDGFRA, platelet-derived growth factor receptor alpha; RET, rearranged during transfection; EGFR, epidermal growth factor receptor; HER2, human epidermal growth factor receptor 2; NTRK, neurotrophic tyrosine receptor kinase; MKI, multikinase inhibitor.

Supplementary Table 7. Clinical benefit according to ESMO–MCBS, reimbursement status, and regulatory outcomes of antineoplastic medicines granted conditional marketing authorisation

Active Substance	ESMO–MCBS Score (*)	Reimbursement for the 1st indication of approval in Spain	CMA approval date	Date of reimbursement decision or withdrawal	Time to reimbursement (years)	Regulatory status (31 Mar 2025)
Alectinib	No ESMO–MCBS score available for the first approval studies**	Reimbursed	16/02/2017	01/04/2018	1.12	SMA
Amivantamab	3	Not reimbursed	09/12/2021	15/12/2025	NA	SMA
Avapritinib	3	Reimbursed	24/09/2020	01/01/2024	3.27	CMA
Avelumab	4	Reimbursed	18/09/2017	01/09/2018	0.95	SMA
Belantamab Mafodotin	No ESMO–MCBS score available for the first approval studies	Withdrawn MA	25/08/2020	23/02/2024	NA	Withdrawn MA
Brexucabtagene Autoleucel	3	Reimbursed (restricted)	14/12/2020	01/02/2024	3.13	CMA
Cemiplimab	4	Reimbursed	28/06/2019	01/04/2022	2.76	SMA
Ciltacabtagene Autoleucel	No ESMO–MCBS score available for the drug	Reimbursed (restricted)	25/05/2022	01/04/2025	2.85	SMA
Dostarlimab	3	Reimbursed	21/04/2021	01/04/2023	1.94	SMA
Entrectinib	3**	Reimbursed (both indications)	31/07/2020	01/12/2023	3.33	CMA

Idecabtagene Vicleucel	No ESMO–MCBS score available for the drug	Reimbursed (restricted)	18/08/2021	01/03/2024	2.54	SMA
Larotrectinib	3	Reimbursed (restricted)	19/09/2019	01/10/2023	4.03	CMA
Loncastuximab Tesirine	3	Not reimbursed	20/12/2022	15/12/2025	NA	CMA
Lorlatinib	4	Reimbursed	06/05/2019	01/02/2021	1.74	CMA
Mosunetuzumab	3	Reimbursed	03/06/2022	01/06/2024	2.00	CMA
Pemigatinib	2	Reimbursed	26/03/2021	01/01/2024	2.77	CMA
Polatuzumab Vedotin	No ESMO–MCBS score available for the first approval studies	Reimbursed	16/01/2020	01/09/2021	1.63	SMA
Pralsetinib	3	Withdrawn MA	18/11/2021	24/10/2024	NA	Withdrawn MA
Rucaparib	3	Reimbursed	24/05/2018	01/01/2020	1.61	SMA
Selinexor	No ESMO–MCBS score available for the drug	Reimbursed	26/03/2021	01/08/2025	4.35	SMA
Selpercatinib	3	Reimbursed (both indications)	11/02/2021	01/05/2023	2.21	CMA
Selumetinib	Not available	Reimbursed	17/06/2021	01/06/2023	1.95	CMA
Sotorasib	3	Reimbursed	06/01/2022	01/01/2024	1.98	CMA
Tafasitamab	3	Reimbursed	26/08/2021	01/05/2023	1.68	CMA
Teclistamab	No ESMO–MCBS score available for the drug	Reimbursed (restricted)	23/08/2022	01/05/2024	1.69	CMA
Trastuzumab Deruxtecan	No ESMO–MCBS score available for	Reimbursed (restricted)	18/01/2021	01/12/2022	1.87	CMA

	the first approval studies					

*Form 3 applied to single-arm response-rate approvals

**Both pivotal studies

“Not available” indicates that no ESMO–MCBS evaluation was available at the time of analysis. “Not available any score for the drug” indicates that the medicine had not been evaluated within the ESMO–MCBS framework. “Not available trial score for approval studies” indicates that an ESMO–MCBS score existed for the drug but not for the pivotal studies supporting the conditional marketing authorisation indication.

Abbreviations: CMA, conditional marketing authorisation; ESMO–MCBS, European Society for Medical Oncology–Magnitude of Clinical Benefit Scale; MA, marketing authorisation; SMA, standard marketing authorisation.
